# The sporting resilience model: A systematic review of resilience in sport performers

**DOI:** 10.3389/fpsyg.2022.1003053

**Published:** 2022-12-21

**Authors:** Sahen Gupta, Paul Joseph McCarthy

**Affiliations:** ^1^Glasgow Caledonian University, Glasgow, United Kingdom; ^2^School of Health Sport and Exercise Sciences, University of Portsmouth, Portsmouth, United Kingdom

**Keywords:** protective resources, athlete, adaptation, resilience response, definition, positive psychology

## Abstract

We systematically reviewed resilience research in sport and exercise psychology. Sample included 92 studies comprising empirical qualitative and quantitative studies, mixed-method studies, review studies and conceptual/theoretical studies on psychological resilience in sports context. From the findings, we synthesized an evidence-based sport-specific definition and meta-model of “Sporting Resilience.” The review incorporates evidence from global culture contexts and evidence synthesized into the new definition and meta-model to achieve its aim. Conceptual detail and testability of the operational definition is provided. Sporting resilience provides a guiding framework for research and applied practice in a testable, objective manner. The new theoretical meta-model of resilience is derived from systematic evidence from sport psychology with theoretical considerations from positive and clinical psychology allowing generalizability. This original theory posits that there is a resilience filter comprised of biopsychosocial protective factors. The strength of this filter determines the impact of adversity and establishes the trajectory of positive adaptation. The findings of the review are used to discuss potential avenues of future research for psychological resilience in sports psychology.

**Systematic review registration:**

https://doi.org/10.17605/OSF.IO/AFWRU.

## Introduction

### What is psychological resilience?

Psychological resilience is the ability to withstand—and/or adapt—after an adversity. Psychological resilience has been studied in high-risk children and communities (Condly, 2006; Mancini and Bonanno, 2009) and among individuals after significant stress producing adversities such as childhood sexual abuse (Bogar and Hulse-Killacky, 2006), death of a parent (Greeff and Human, 2004), and terrorism (Bonanno et al., 2007). Resilience is often termed as “ordinary magic” (Masten, 2001, p. 227) because resilience in children can be developed by the correct combination of environments, relationships, and the chance to explore the world around with psychological safety (Masten, 2001).

### Psychological resilience in sport

Individuals who participate in sport actively engage with failure and adversity. Athletes experience failures, adversities, and stressors of different magnitudes in their careers (Mellalieu et al., 2009; Tamminen et al., 2013). Literature shows that athlete and non-athlete populations experience different stressors (Pritchard and Wilson, 2005), have different body image conceptualization (Hausenblas and Downs, 2001) and show differences in emotional intelligence and mental health (Bostani and Saiiari, 2011). Division 47 of the APA noted that “the sport context is a unique performance environment that requires specialized training beyond general performance principles… because of the unique culture of sport” (American Psychological Association, 2011, p. 14). Besides natural life stressors, athletes also experience obstacles such as injuries (Podlog and Eklund, 2006) and mental health issues (Papathomas and Lavallee, 2012) because of being in a highly evaluative environment with high impact positive and negative consequences associated with outcomes (see for review Sarkar and Fletcher, 2014).

Resilience has been implied as a functional necessity for success in sport because “the question is not *if* an athlete will encounter adversity in sport, but instead *how* will they respond when adversity occurs (Galli and Gonzalez, 2015, p. 1, italics as in original). Fletcher and Sarkar (2012) used grounded theory to study Olympic champions (experiences of adversity) and formulated the first sport-specific definition of psychological resilience: “the role of mental processes and behavior in promoting personal assets and protecting an individual from the potential negative effect of stressors” (p. 675).

Narrative reviews have outlined the stressors (e.g., performance standards, selection, funding, injury, media evaluations) and protective factors of psychological resilience (e.g., social support, environment, metacognitive appraisal) in sport performers (Sarkar and Fletcher, 2014) as well as implications for research and practice (see Galli and Gonzalez, 2015). Narrative reviews provide excellent evidence-based insight and a historical overview, but are difficult to replicate (Pae, 2015). In contrast, a systematic review adopts a structured, replicable method to search and analyse literature on a topic, providing insights into the empirical/theoretical advancements in an area (Hanley and Cutts, 2013).

A citation network analysis by Bicalho et al. (2020) indicated that there has been a rapid increase of publications on resilience between 2012 and 2018. A systematic review conducted in 2016 presented the definitions of resilience used in literature and the relationship of resilience with other psychological resources (see Bryan et al., 2019). Therefore, there is a need to review the resilience literature because previous narrative reviews preceded the expansion of publications. A systematic review categorizes and catalogs evidence across multiple studies to provide reliable findings with observable conclusions (Chandler et al., 2017, p. 5). This systematic review finds its rationale in an updated summary of the evidence base and future directions for research.

### Resilience: Conflations

A review that summaries existing literature is crucial because previous evidence indicates that there are instances of ambiguous theorizing which hamper the understanding of resilience (Bryan et al., 2019). This imprecision creates simplistic “colloquialisms” in applied practice (p. 70). An updated systematic review of recent literature clarifies and guides future research. Researchers often conflate resilience or use it interchangeably with coping (Campbell-Sills et al., 2006; Rutter, 2012), mental toughness (Gucciardi et al., 2011), hardiness (Windle, 2011; Howe et al., 2012), and thriving (Brown et al., 2020). For instance, resilience is the process of adaptation post exposure to adversity/stressors (Luthar et al., 2000; Fletcher and Sarkar, 2012; Sarkar and Fletcher, 2013) whereas thriving is a value-added construct which describes the process of achieving a greater level of functioning in response to threat and risk (O'Leary and Ickovics, 1995).

Therefore, while resilience characterizes adaptive recovery (i.e., return to pre-adversity level of functioning by adaptation), thriving is value-added (i.e., exhibition of a superior level of functioning) (see Carver, 1998; Brown et al., 2020). Similarly, mental toughness, defined as “unshakeable perseverance and conviction toward some goal despite pressure or adversity” (Middleton et al., 2004, p. 1) is distinct from resilience. To illustrate, resilience in sport performers arises out of protective factors (see Sarkar and Fletcher, 2014). Resilient individuals can engage these protective factors to adapt successfully to adversity and stressors (Waaktaar and Torgerson, 2010; Windle, 2011; Fletcher and Sarkar, 2013); however, empirical evidence often strays from this operationalisation (Gucciardi et al., 2011). For example, Estrada et al. (2016) noted that 89% of the measures of resilience indirectly measuring antecedents, outcomes and/or covariates of resilience, not resilience itself. The current systematic review will (1) synthesize and summarize the growing evidence base to display which definitions of resilience studies are using, (2) appraise the definitions used to check whether they are supported by the empirical evidence, and (3) provide an operational definition of sporting resilience supported by the evidence from the systematic review.

### What does this study do?

Bryan et al. (2019) argued for defining resilience accurately using evidence from peer-reviewed research. Evidence-based definitions are essential to embrace sound scientific standards of research and rigorous applied practice (Moore, 2007; Winter and Collins, 2015). This article presents a systematic review of research that included resilience as a direct variable of investigation. We extend the conceptual ideation put forward by Den Hartigh et al. (2022) by using a systematic review method to isolate trends in the resilience in sport evidence base. There are four objectives of this study: first, we summarize the current empirical evidence base, and the definitions of resilience used. Second, we extract data from the empirical evidence to evaluate the definitions of resilience in sport for relevance. Third, we review the evidence to understand which empirical findings support which aspects of resilience theory. Four, reviewing theory present in literature against recent empirical evidence, we deliver a focused investigation into each component of resilience in the sporting context and develop the proposed meta-model.

## Methods

### Frameworks and procedure

This systematic review used Pluye and Hong (2014) and PRISMA (Moher et al., 2015) models for systematic reviews to best extract, appraise and synthesize data on resilience research in sport. This combination is replicated here because it has been used to systematically review sport psychology literature (cf. Gledhill et al., 2017; Bryan et al., 2018). This review was registered in the Open Science Framework for transparency, reproducibility and reduction of potential bias. All data related to the review and registration is available at (https://osf.io/afwru/?view_only=ab1ff15d3fff4bc18a96f6a011cfbe84). This review is integrative (collating different sources of data on resilience) and inductive (observations from analysis of existing research is appraised to come up with a general principle). We provide a synthesis of evidence of empirical, review and conceptual literature on resilience in sport.

### Eligibility criteria

Studies were selected in line with the inclusion criteria of: (a) original peer-reviewed articles; (b) book chapters because they are a valuable source of theoretical discourse; (c) full-text was obtainable; (d) examined psychological resilience at the individual level in sport contexts; (e) empirical studies that examined protective factors of psychological resilience or outcomes of being resilient in sport as a variable of investigation.

Studies that operationalized psychological resilience as stress-related growth and/or mental toughness and/or adversarial growth (see section above for resilience operationalization) were deemed ineligible to ensure clarity and a superior answer to the research question. Unpublished literature was excluded because they typically have no abstract and search markers to match against inclusion criteria (Benzies et al., 2006; Pappas and Williams, 2011). Review literature was included since they show conceptual development and inference of evidence into theory across the history of sport resilience research. Non-English literature was excluded due to lack of English translation resources; however, this exclusion does not constitute a limitation to the global nature of this systematic review because many non-Western countries have active English-publication scientific communities.

### Research strategy

We conducted an initial scoping search from 1st to 5th November 2020 to check the feasibility of the review. A later search was conducted on May-June 2021. Final updated search was conducted from 20th December 2021 to 5th January 2022 using the strategy outlined below.

#### Search strategy

**Figure 1:**
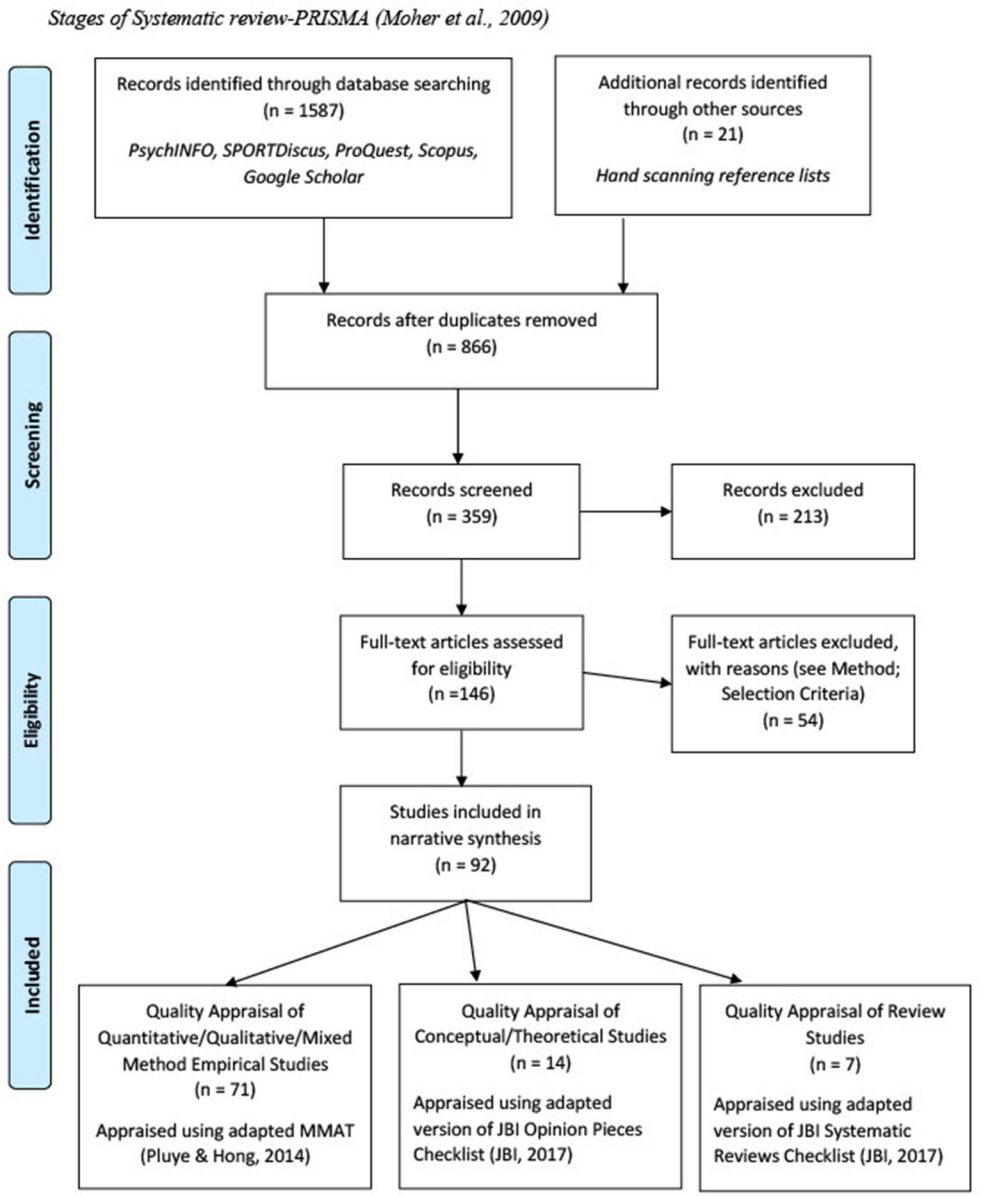
Stages of systematic review-PRISMA (Moher et al., 2015).

The search was conducted using the following integrated combination of keywords as Boolean operators to search for titles and keywords: Resil^*^ AND athlete^*^ AND Success; Resil^*^ AND Sport; Resil^*^ AND Coach; Resil^*^ AND Sport^*^; Bounce^*^ AND Back^*^ AND Sport^*^; Resil^*^ AND Player^*^. Electronic databases of PsychINFO, SPORTDiscus, ProQuest (Nursing and Allied Health Database; Sports Medicine and Education) and SCOPUS were searched. Further double-checking searches using paper titles and keywords using Google Scholar and ResearchGate was conducted to ensure relevant papers were not excluded. Reference sections of retained papers were hand-scanned to ensure thorough search of literature (Greenhalgh and Peacock, 2005). Authors were emailed to secure full texts if not available *via* libraries. A study was excluded if authors did not respond after three email contacts. No publication limit was set to capture all relevant evidence on resilience research in sport in line with the research question. In total, this search produced 1,598 studies (see Figure 1).

#### Data management

Searches were collated, noted, and traced manually using Microsoft Excel. De-duplication from databases was done in Excel and crosschecked using RefWorks. Assessment for inclusion was done in two levels. First, title and abstracts were screened against inclusion criteria (Level I). Where screening could not be undertaken by abstract alone, full-text was screened against inclusion criteria (Level II). The process is highlighted in the PRISMA flow diagram (Moher et al., 2015) (see Figure 1). The second author independently carried out the same search for rigor. Discrepancies were discussed and accepted/rejected according to eligibility criteria. Ten percent of the included articles were randomly selected for an independent third party peer review to confirm the rationale for including articles according to the pre-set criteria. The lead author rather the quality of method used in the study before reading results section to prevent bias while doing full-text analysis (Higgins et al., 2019). The second author reviewed a random selection of the quality appraisal to ensure rigor.

#### Data extraction and quality appraisal

Data extraction from the selected studies was conducted using an extraction protocol which focused on (i) Conceptual/Theoretical framework of resilience; (ii) Operational definition of resilience used; (iii) Method with focus on design, sample (with demographics, sport type, sport level) and analysis procedures; (iv) Measures used to study resilience; (v) Results. Data extracted from included studies were manually recorded. This was then uploaded into a spreadsheet for quality appraisal and synthesis prior to transfer to tables during manuscript writing. Methodological quality for empirical studies was assessed using an adapted MMAT framework (Pluye et al., 2009; Pluye and Hong, 2014). For systematic reviews and conceptual papers/chapters, an adapted version of the JBI Systematic Review and Opinion Pieces checklist (Joanna Briggs Institute, 2017) was used (see Appendix A). Total number of questions were converted to provide a percentile point of 100. “Yes” responses to each question gave an equal point weightage score. “No” responses equalled zero (see Appendix A for assessment tool). The second author extracted data using the protocol from a random selection of articles. If independently extracted data was discrepant. Authors reverted to the original article and discussed to clarity to achieve consensus.

#### Data synthesis

A theoretical synthesis of findings was conducted following data extraction and quality appraisal replicating best practice guidance in systematic reviews (see cf. Pluye and Hong, 2014; Moher et al., 2015; Gledhill et al., 2017; Bryan et al., 2019). Theoretical synthesis process included: (a) Understanding resilience construct placement (i.e., whether resilience was viewed as a “trait” i.e., stable and difficult to change or “dynamic” i.e., malleable with conditions); (b) Theoretical orientation (i.e., theoretical frameworks used to understand resilience in selected studies); (c) Appraisal of empirical evidence to see which components of resilience construct and theory is supported/refuted. Meta-analysis was not conducted since data included multiple research designs.

## Results and discussion

### Synthesis of included research

The final sample comprised 92 studies. Seventy-one were empirical studies (quantitative = 54; qualitative = 13; mixed methods = 4), 12 were theoretical/conceptual studies and 7 were review studies. The overall quality appraisal score overall was high at 85.22%. Empirical studies scored at 83.03% (Quantitative = 76.59%; Qualitative = 98.66%; Mixed-Methods = 90%), theoretical/conceptual studies were scored at 98.66% and review studies were scored at 86.85% (see Appendix B).

The process of theoretical synthesis was conducted in two phases and is outlined to enable replication. In Phase 1, data (i.e., articles) were analyzed by clustering variables that were linked to resilience by frequency counts. For example, “Mastery”/“Sense of Control” was explored by 6 studies. In Phase II, the relevant studies exploring a variable of interest (e.g., “Mastery”/“Sense of Control”) was analyzed to infer whether the empirical evidence in those studies indicated that mastery was a key protective factor of resilience. If the empirical evidence indicated so, the variable was incorporated into the theoretical synthesis. The frequency count and empirical studies for each variable and/or characteristic of resilience is outlined in Tables 1, 2 for rigor and replicability.

**Table 1:** Definitional clarity and empirical evidence of components of “sporting resilience.”

**“Sporting resilience” components for testability**	**Definitional clarity**	**#Frequency -> Empirical evidence**
Dynamic	Resilience is a process that is characterized by constant change through interactions between risk and protective factors (Rutter, 2012)	#7-> (Luthar et al., 2000; Bryan et al., 2018; Galli and Pagano, 2018; Hill et al., 2018a, 2020; Blanco-García et al., 2021; Gupta and McCarthy, 2021)
Environmentally adaptable	Fluidity in the face of changing environmental conditions (sporting and general life) that shape antecedents and consequences of resilience to ensure positive adaptation i.e., “if circumstances change, resilience alters” (Rutter, 1981, p. 317).	#5 -> (Rutter, 1981; Sarkar and Fletcher, 2014; White and Bennie, 2015; Fletcher and Sarkar, 2016; Wagstaff et al., 2016).
Interaction-dominant	Active interface characterized by inter-individual (individual and environment) and intra-individual (individual and protective resources) functional interaction over time.	#3 -> (Den Hartigh al., 2018; Hill et al., 2018b, 2020)
Process-trajectory	Resilience as a process is unfolds through the relative path/trajectory determined by the individual's resources and adversity experiences that occur in isolation or concurrently	#4 -> (Brown et al., 2015; Fletcher and Sarkar, 2016; Gupta and McCarthy, 2021)
Metacognitive capacity	The capacity to engage in a search for insight into and control over one's own mental processes (Flavell, 1979) such as explanatory style, perceived competence, self-concept/insight, beliefs contributing to better mobilize resources for resilient adaptation.	#11 -> (Galli and Vealey, 2008; Fletcher and Sarkar, 2012; Secades et al., 2016; Brown et al., 2020; cf. explanatory style Seligman et al., 1990; Martin-Krumm et al., 2003; cf. perceived competence see Machida et al., 2013; Vitali et al., 2015; cf. self-concept Zurita-Ortega et al., 2016; cf. self-insight Cowden and Meyer-Weitz, 2016; cf. beliefs Ripley, 2008),
Emotional capacity	The capacity to be aware of and engage one's emotional reactions intelligently and appropriately avail positive emotions in adversity situations to broaden and build thought-action trajectories (Fredrickson, 2004)	# 11 -> (Fredrickson, 2004; Galli and Vealey, 2008; Chandler et al., 2020; cf. meaning/belonging Smith et al., 1990; Hall, 2011; Meggs, 2016; cf. positive meaning Hall, 2011; Timm et al., 2017; Codonhato et al., 2018; Trigueros et al., 2019; Madsen et al., 2021)
Behavioral capacity	The capacity of an individual to perform behaviors through knowledge and skills that allow a positive reciprocal relationship between behavior and environment conferring resilience.	#9 -> (Belem et al., 2014; cf. “adaptive trio” Yi et al., 2005; cf. locus of control Zurita-Ortega et al., 2018; cf. self-determination Subhan and Ijaz, 2012; cf. self-regulation Mummery et al., 2004; Belem et al., 2014; Gupta and Sudhesh, 2019; Kegelaers et al., 2019; Trigueros et al., 2020a)
Equilibrium and positive adaptation	The state of resting balance due to equal opposite forces of negative adversity and positive protective resources characterized by adaptation to adversity and return to pre-adversity levels of functioning.	#2 -> (Bonanno and Diminich, 2013; Hill et al., 2018b) + Novel Conceptualization of this Study

**Table 2:** Definitional clarity and empirical evidence of components of “biopsychosocial protective filter” of sporting resilience.

**“Biopsychosocial protective filter” components**	**Definitional clarity/measurement tools**	**#Frequency -> Empirical evidence**
Perceived/tangible social support	The perception of and/or the actuality that an individual has the provision of assistance in the form of emotional/psychological support, informational and tangible support. Can be measured by the PASS-Q (Freeman et al., 2011)	#21 -> (Holt and Dunn, 2004; Mummery et al., 2004; Yi et al., 2005; Galli and Vealey, 2008; Hall, 2011; Fletcher and Sarkar, 2012; Morgan et al., 2013, 2015, 2019; Brown et al., 2015, 2020; Cox et al., 2016; Lu et al., 2016; Yamada et al., 2017; Codonhato et al., 2018; Adam and Cogan, 2019; Drew and Matthews, 2019; Aydogan and Gaye, 2020; Chandler et al., 2020; Trigueros et al., 2020b; Sullivan et al., 2021)
Motivation/motivational climate	• The psychological climate of the sporting environment that is curated by the coach and/or organization that enhances motivation in training and competition (adapted from Ames, 1992) • Could be measured by Perceived Motivational Climate in Sport Questionnaire (Walling et al., 1993)	#8 -> (Subhan and Ijaz, 2012; Machida et al., 2013; Codonhato et al., 2018; Chacón-Cuberos et al., 2019; Trigueros et al., 2020a; cf. Martin et al., 2015; Pedro, 2016; Athlete engagement González et al., 2019)
Metacognitive-challenge appraisal	• Processes utilized to plan, monitor, and assess adversity as challenging and having adequate ability and personal resources to grow and master from adversity experience. • Could be measured by Metacognitive Processes During Performances Questionnaire (Love et al., 2019)	#16-> (Seligman et al., 1990; Martin-Krumm et al., 2003; Schinke et al., 2004; Galli and Vealey, 2008; Fletcher and Sarkar, 2012, 2013; Machida et al., 2013; Cardoso and Sacomori, 2014; Vitali et al., 2015; Pedro, 2016; Secades et al., 2016; Deen et al., 2017; Galli and Pagano, 2018; Adam and Cogan, 2019; Brown et al., 2020; Trigueros et al., 2020a)
Sense of meaning/belonging	• The individual's sense of meaning and emotional need of belonging central to their sense of “self,” their sport and its expression in their personal/sporting life. • Can be measured by Perceived Belonging In Sport Scale (Allen, 2006)	#11 -> (Smith et al., 1990; Hall, 2011; Martin et al., 2015; Meggs, 2016; Timm et al., 2017; Codonhato et al., 2018; Adam and Cogan, 2019; González et al., 2019; Aydogan and Gaye, 2020; Trigueros et al., 2020a)
Self-regulation ability	• The ability to understand, manage and control one's thoughts/emotions/behavior disruptive to the pursuit of their short- and long-term goals. • Can be measured by Emotional Regulation Questionnaire (Athletes) (Uphill et al., 2012) and Self-Regulation Questionnaire (Carey et al., 2004)	#6 -> (Belem et al., 2014; Fletcher and Sarkar, 2016; Gupta and Sudhesh, 2019; Kegelaers et al., 2019; Trigueros et al., 2020a)
Mastery/sense of control	• Broadly defined as mastery and sense of control over one's life circumstances within and outwith of sport. • Can be measured by Sense of Agency Scale (SoAS) (Tapal et al., 2017)	#7-> (Galli and Vealey, 2008; Fletcher and Sarkar, 2012, 2016; Morgan et al., 2013; Pedro, 2016; Zurita-Ortega et al., 2018; Gupta and McCarthy, 2021)
Optimism	• The attitudes reflecting a sense of hope and belief that outcomes of specific actions will be favorable, desirable, and positive. • Can be measured by Personal Optimism Scale or Self-Efficacy Optimism Scale (Gavrilov-Jerković et al., 2014)	#4 -> (Young, 2014; Codonhato et al., 2018; Kegelaers and Wylleman, 2019; optimistic coping- Özyurt Kiliç, 2021)
Facilitative environment	• The physical and psychological sporting environment of the individual which adequately balances challenge and support to optimize positive growth, performance and resilience (Fletcher and Sarkar, 2016; Sanford). • Can be measured by tracing self-report qualitative responses on the Challenge-Support Matrix (Fletcher and Sarkar, 2016)	#8 -> (Fletcher and Sarkar, 2016; Galli, 2016; Pedro, 2016; Wagstaff et al., 2016; Sarkar, 2017; Adam and Cogan, 2019; Drew and Matthews, 2019; Trigueros et al., 2020b)
Passion/love of sport	• Passion is defined to be the strong inclination toward sport as a self-defining activity that is loved, important to and in which the individual invests time and energy on a regular basis (adapted from Vallerand, 2008). • Can be measured by the Two-Factor Passion Scale (Marsh et al., 2013) or through qualitative triangulation data	#7 -> (Galli and Vealey, 2008; Machida et al., 2013; Brown et al., 2015, 2020; Timm et al., 2017; Codonhato et al., 2018; Aydogan and Gaye, 2020)
Identity/self-insight	• The qualities, beliefs, expressions, standards i.e., the mental model of the sporting individual's “self” that is developed through introspection. • Can be measured through motivational interviewing or through triangulation qualitative data	#6-> (Mummery et al., 2004; Cowden and Meyer-Weitz, 2016; Zurita-Ortega et al., 2016; Trigueros et al., 2019; Brown et al., 2020; Gupta and McCarthy, 2021)

### Resilience in sport: A construct with definitional variety

Results of this systematic review provide fruitful insight into the definitional heterogeneity of resilience research in sport. We augment the preliminary findings of Bryan et al. (2019) by recognizing there are multiple definitions of resilience. Most definitions are “borrowed” from other fields of psychology and are not validated in the sport context. Among the included studies, 66 cited 25 guiding definitions of resilience. Nine outlined their own definition, 21 provided no operational definition (summarized in Appendix C). Multiple corresponding definitions were found. The definition by Fletcher and Sarkar (2012) is from the sport context but is restricted to Olympic champions and may not be ecologically valid. Few other definitions conceptually review sport as a setting (see Galli and Pagano, 2018; Hill et al., 2018a,b).

Bicalho et al. (2020) noted that 60% of studies on psychological resilience in sport since 2012 used Fletcher and Sarkar's (2012) definition. Results of our systematic review note that 22.8% of the included articles use this definition. Although this definition provides an excellent foundation, analysis indicates several areas where research can evolve to refine conceptual and methodological clarity. First, they operationalise resilience as psychological and note it to be “the role of mental processes and behavior in promoting personal assets and protecting an individual from the potential negative effect of stressors” (p. 675); however, resilience is a dynamic process which does not stop at protection from stressors, but encompasses positive adaptation (Luthar et al., 2000, p. 543; Luthar, 2006; Hill et al., 2018a; Gupta and McCarthy, 2021) not only from stressors/adversities but also from novel challenges in new situations, because if “circumstances change, resilience alters” (Rutter, 1981, p. 317). A major strength of the definition is the inclusion of mental processes and metacognitive components; however, it does not consider the developmental component of resilience because it is a capacity that develops over time in relation to the context of person-environment interactions (Egeland et al., 1993; see for definitional review Fletcher and Sarkar, 2013). Resilience exists on a continuum present to different degrees in different contexts (Pietrzak and Southwick, 2011), with specific influence of environmental and sociocultural contexts (Wagstaff et al., 2016).

Bryan et al. (2019) conducted a frequency word analysis on guiding definitions of resilience and noted that most definitions included three core concepts: adversity, positive adaptation, and bouncing-back/rebound and maintenance of wellbeing in line with Fletcher and Sarkar (2013). Synthesizing the frequency analysis of the most “prominent and frequent aspects of a multitude of definitions” (p. 77) they provided a definition stating resilience to be “a dynamic process encompassing the capacity to maintain regular functioning through diverse challenges or to rebound using facilitative resources” (p. 77). This definition is classifying resilience as a dynamic process, where individuals use resources to rebound after adversity (Sarkar and Fletcher, 2014). However, using a frequency word count of existing definitions to create another definition is not an empirically based conceptualization. This definition is synthesized from work and sport literature and is not tailored to the sport context. This point is crucial because resilience is best understood within domain-specific contexts (Luthar and Cicchetti, 2000; Fletcher and Sarkar, 2013). Since it is partially founded upon a work context, the definition does not account for the unique environmental configurations of the phenomenological reality of sport.

Hill et al. proposed a dynamical perspective of resilience in sports (Hill et al., 2018a) and a definition (Hill et al., 2018b) noting resilience to be “the dynamic process by which a biopsychosocial system returns to the previous level of functioning following a perturbation caused by a stressor” (p. 367). This “biopsychosocial system” is a conceptual advancement because the sports setting is a complex amalgam of physiological capacity, psychomotor skills, psychological elements, and social processes. They do not, however, provide an empirical backing to the conceptualization. Resilience is conceptualized as an outcome of withstanding perturbations and returning to a previous state. And this excludes positive adaptation capacity, which is a cornerstone of resilience operationalization distinguishing it from hardiness and/or mental toughness (see Gucciardi et al., 2008; Windle, 2011).

Clarity is crucial because “concepts are integral to every argument, for they address the most basic question of social science research: what are we talking about?” (Gerring, 2012, p. 112). We argue that the sport context is unique where athletes encounter multiple challenges/adversities simultaneously rather than in temporal isolation (Galli and Reel, 2012). Kiefer et al. (2018) also highlighted the importance of studying resilience as a situated, iterative-process driven by multiple variables whose influence is time-dependent and contextual. Therefore, operationalization of resilience needs to be contextually specific (cf. Luthar and Cicchetti, 2000; Fletcher and Sarkar, 2016; Wagstaff et al., 2016; Sarkar, 2017), founded upon empirical literature from sport psychology. The results of this systematic review provide the rationale to conceptualize a “sporting” i.e., sport-context specific model. The individual in the sporting context is not socially isolated, and therefore, by logical extension, nor is their resilience. Rather, because of their involvement in the sporting context, resilience is formed from and used to maintain positive equilibrium and/or adapt to a diverse range of sport-related stressors (see Fletcher and Sarkar, 2012; Gupta and McCarthy, 2021).

### “Sporting resilience”: An operational definition

Keeping in mind the limitations of existing definitions and sourcing empirical evidence from this systematic review, we propose a definition of sporting resilience. The definition does not pull together broad descriptors but collates components which have found empirical support. We adhered to recommendations from literature to limit subjectivity (Gerring, 2012; Goertz and Mahoney, 2012).

Our definition outlines “*Sporting resilience is a person's ability to evaluate what they think, feel and do when faced with an adversity which allows them to operate at their previous level and successfully adapt to persist*.” Sporting resilience is learned as a process through interactions with the world (see Table 1 for components and evidence synthesis). Evidence indicates that sporting resilience is the environmentally adaptable, interaction dominant, dynamic-process trajectory that encompasses a sporting individual's metacognitive-emotional-behavioral capacities to maintain a positive equilibrium and successfully adapt to a diverse range of sport-related adversities. Although sporting resilience captures an individual's resilience process in sport, it also is learned from non-sport components because individuals do not live in a vacuum.

The definition of sporting resilience is a comprehensive, empirically deduced definition by considering all aspects of the ontology of resilience in sport rather than reduction in favor of convenience (Podsakoff et al., 2016). The definition encompasses capacities, processes, and outcomes in line with recommendations of a constructivist, holistic conceptualization for multidimensional concepts (Blalock, 1968; MacKenzie et al., 2011). Empirical findings were systematically reviewed to formulate the final definition in evidence-based antecedents and consequences (Podsakoff et al., 2016) (see evidence mapping in Table 1). Resilience is a multidimensional construct that manifests in protective factors and outcomes (Fletcher and Sarkar, 2013). Our definition comprehensively outlines the sporting resilience construct, with its constituent components of the definition open to empirical verification. This serves to circumvent ambiguity fallacy (Bennett, 2012). For example, empirical research can study the “interaction dominant” component *via* empirical testing with controls to see whether resilient individuals gain from interaction. The comprehensive and concrete elements of the definition are isolated from existing empirical support (see Table 1) which will aid future operationalisations of resilience to measure and test specific components of the observed reality of sporting resilience. The range of testable components proposed by the definition heeds William James' warning of vicious abstractionism which “becomes a means of arrest far more than a means of advance in thought” (James and Katz, 1975, p. 136).

#### Conceptual detail and testability

Table 1 highlighted above provides the definitional clarity and empirical evidence of each component of the definition of sporting resilience. We also provide conceptual detail from the evidence synthesis to provide clarity for testability and operationalization of the definition.

**Figure 2:**
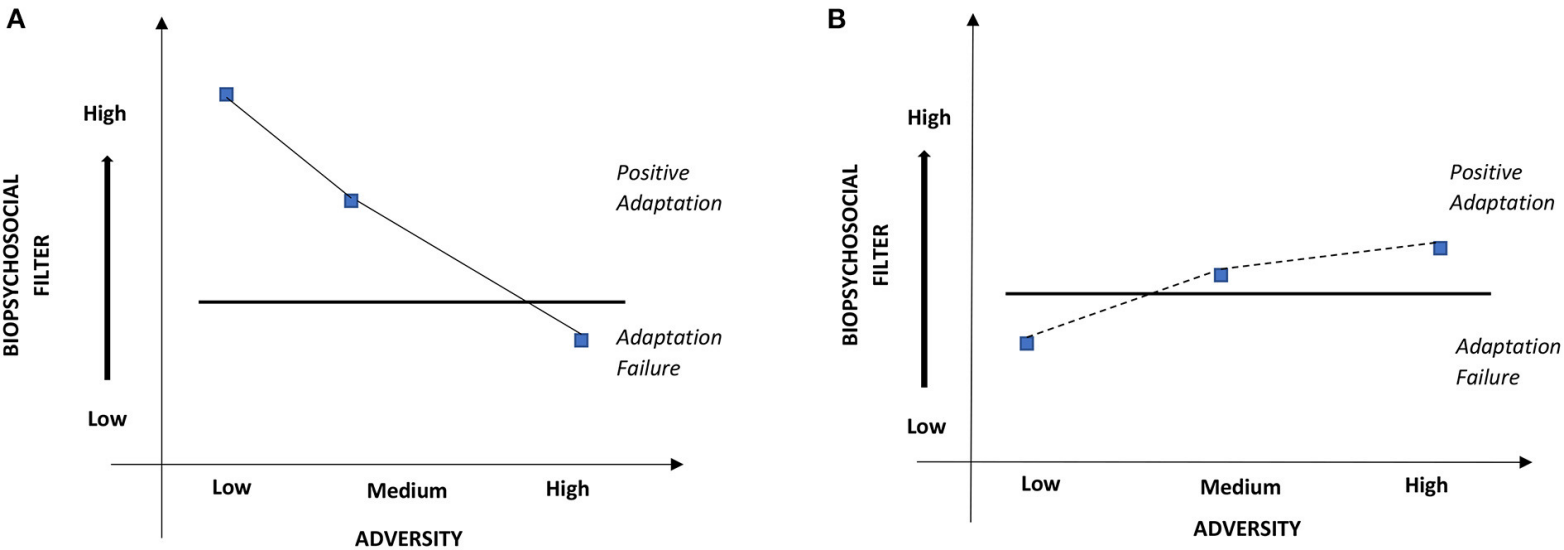
Graphical model of adversity adaptation. Two examples of relationship between biopsychosocial filter strength, adversity intensity and resilience process. The left graph [Athlete **(A)**] is an example of an athlete with various levels of protective resources interacting with adversities of different intensities. Athlete **(A)** engages resilience and positively adapts when filter is strong enough to handle adversity and has adaptation failure when high-intensity/acute adversity becomes too much for low biopsychosocial resources. The right curve [Athlete **(B)**] is an example of a resilience response engaged over time relative to a matched adversity-biopsychological filter level where the athlete is engaged in a prolonged and iterative learning process. In the case of Athlete **(B)**, this is an example of emergent process-trajectory of resilience (hypothetical scenario mapped for pictorial representation.

- Sporting resilience is defined as “dynamic” because it is changing and is determined by temporal and interactive factors such as a moment in one's career, personal life circumstances, and nature of adversity colloquially characterized as “ups and downs” “when-what-where.” There is a constant interface between the individual and the environment, making sporting resilience “environmentally adaptable.” For instance, Fletcher and Sarkar (2016) strongly advocated how an environment providing balanced challenge and support contributes to building resilience.
- Sporting resilience is “interaction-dominant” which means it is determined by the interaction of an individual within themselves and engaging with their environments, resulting in a dynamic cycle of environmentally adaptable learning and relearning. Behavior patterns emerge and alter over time as the athlete with existing capacities interact with an ever-evolving environment resulting in a change (Hill et al., 2018a). This interactional learning occurs in sport and non-sport environment; however, an emphasis is on the sport environment because individuals spend the bulk of their time in that context.
- We hypothesize that the “dynamic” nature of sporting resilience is mediated by its “environmentally adaptable” and “interaction-dominant” components. Sporting resilience arises from, and in response to, sport-related adversities (Sarkar and Fletcher, 2013). We propose it is an iterative learning process transferrable in line with qualitative evidence from Hall (2011), which notes how resilience was something athletes had taken from sport to general life. Non-sport experiences also play an active role in the dynamic developmental process of sporting resilience. For example, dealing with race/sex/ethnic based discrimination could result in dynamic action taken by the individual to forge a self-identity, perceived/tangible social support and/or a motivational climate which would have a transfer and develop resilience to be used in sport (Fletcher and Sarkar, 2016; Wagstaff et al., 2016).
- Sporting resilience has a dynamic process-trajectory. It maximizes performance and adaptation capacity while adhering to a set of constraints determined by one's protective factors. The sport performer chooses context appropriate solutions by engaging their protective resources in an environmentally adaptable manner, ensuring performance and positive adaptation (Davids et al., 2013). This concept of “metastability” of resilience grants the ability for environmental-appropriate creative task solution which enables positive adaptation to adversity (Kiefer et al., 2018). The process is a trajectory (i.e., constrained by the extant protective resources that the individual has and must creatively use to adapt). For example, cricket batsmen who faced performance slumps avoided putting a label of “out-of-form” on his slump. He then engaged available personal resources such as work ethic, confidence and viewed “slumps as opportunities for personal growth and learning” (Brown et al., 2020, p. 284) (see Figure 2).
- The existing protective resources determine the process-trajectory which includes “metacognitive-emotional-behavioural” capacities. These capacities operate in tandem and not in isolation because cognitive evaluation of thinking, emotional responses and behavioral capacities often have high overlap (see CBT models Beck and Beck, 2011; Padesky and Mooney, 2012; Turner, 2016, 2019). These capacities develop over time through repeated adversity experiences. They are influenced by the individuals sporting and personal life experiences. For example, from an REBT perspective, resilience comprises flexible cognitive-emotive-behavioral responses to adversities which can be learned (Turner, 2016; Deen et al., 2017). We often see this through the ability to monitor, assess and replace debilitative negative thoughts with facilitative positive ones (i.e., cognitive reappraisal/flexibility) (Wu et al., 2013; McRae and Mauss, 2016).

The breakdown of the definition of sporting resilience makes it operational. The dynamic nature of resilience is rooted in dynamic psychological processes, but clinical assessors such as psychometrics capture snapshots at a moment in time or retrospective, or aggregated over time (Wright and Hopwood, 2016). Assessment and formulation principles from CBT and REBT, which aim to secure quantitative/qualitative/situational information on “who-what-where-when” shows promise. Psychometrics used as part of mixed-method, longitudinal designs have already shown promise in evaluating resilience (see Kegelaers et al., 2019; Chandler et al., 2020). Qualitative evidence in extant literature rates highly and has provided insight into the dynamic process of resilience across various sport samples and contexts (see Fletcher and Sarkar, 2012; Brown et al., 2015, 2020; Morgan et al., 2015, 2019; Timm et al., 2017). These techniques would also provide insight into the “metacognitive-emotional-behavioural” capacities that the individual possesses. Motivational interviewing also holds relevance as a source of testability (Mack et al., 2017) particularly if underpinned by self-determination theory (Markland et al., 2005). Maintenance of equilibrium and positive adaptation to adversity are relatively easy to determine because they are commonly overtly observed or can be sourced by triangulation observational data and inputs from the athlete and others in the sporting environment.

### Theoretical integration into a “sporting resilience meta-model”

Three sport-specific theories of psychological resilience materialized in this review: conceptual model of psychological resilience (Galli and Vealey, 2008), grounded theory of psychological resilience (Fletcher and Sarkar, 2012) and team resilience theory (Morgan et al., 2015). Empirical studies in sport have used non-sport theoretical models such as the resiliency model (Richardson et al., 1990), process conceptualization of resilience (Luthar et al., 2000), challenge model of resilience (Fergus and Zimmerman, 2005) and self-determination theory (Deci and Ryan, 2012). There are also recent conceptual models, such as the dynamic perspective of resilience in sport (Hill et al., 2018a) which has sparked response commentaries (cf. Galli and Pagano, 2018; Hill et al., 2018b) (see Appendix B for overview).

**Figure 3:**
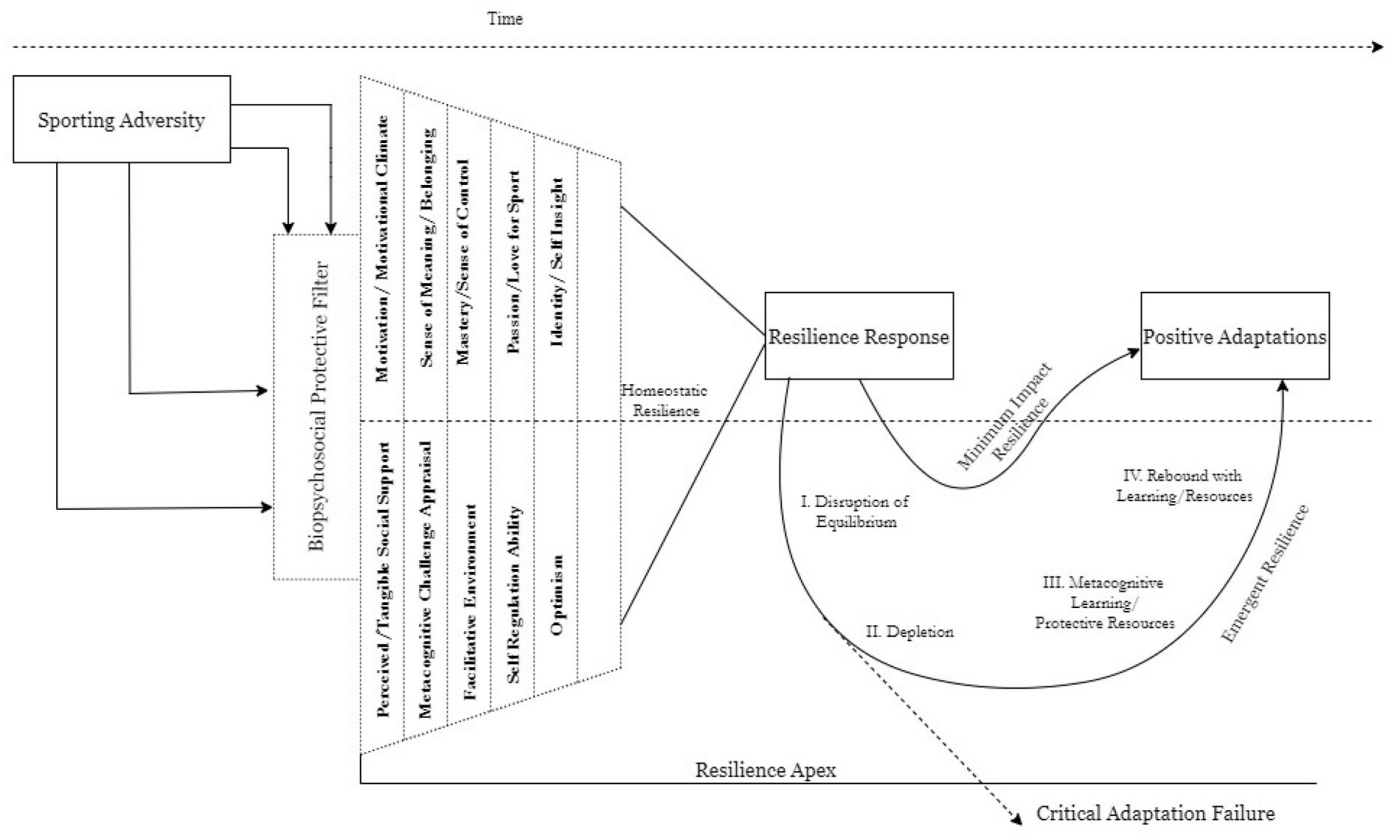
Sporting resilience meta-model. The adversity experience passes through the individual's biopsychosocial protective filter. Depending on the strength of the resources available in this protective filter, the impact of the adversity initiates a specific trajectory of resilience response. Response Trajectory A- causes a minimum disruption due to low intensity adversity and/or a strong protective filter causes quicker resilience adaptation. Trajectory B- undergoes through four levels of Disruption of Equilibrium, Disruption, Metacognitive Appraisal and Rebound during the course of resilience adaptation.

There is a theory-practice gap in sport psychology associated with transferring research into applied practice (Vealey, 2006; Keegan et al., 2017). The sharp growth of resilience research in a brief span of time (2012–2020) is at risk of becoming fragmented and suffers from the same theory-practice gap. The empirical findings of this systematic review update the theoretical conceptualisations of resilience that have empirical support. Aligned to the assertions of Den Hartigh et al. (2022), we build upon existing theoretical formulations of protective factors and resilience response to provide an evidence based conceptual advancement in line with theory development research (Magee, 1974). This integration equips researchers and practitioners with a testable framework for resilience for research and practice. Synthesizing the existing evidence (see Tables 1, 2) (cf. Appendix C), we propose the meta model of sporting resilience (see Figure 3).

The model results from a synthesis of empirical evidence. Many of the components operate concurrently and subjectively in an individual's phenomenological reality. The meta model outlines the protective factors of resilience that have received empirical support. In line with the inductive approach of this theoretical synthesis (see Jones et al., 2009 for precedence), we first reviewed the empirical evidence. Studies which provided quality evidence regarding a specific variable acting as a protective factor of resilience were included as part of the “biopsychosocial filter.” The list does not imply that every individual has all those protective factors. Rather, it highlights the idiosyncrasy of the resilience process determined by the dynamic person-environment-adversity interaction (Luthar et al., 2000; Galli and Pagano, 2018; Hill et al., 2018a; Bryan et al., 2019).

In line with Galli and Reel (2012), guided by recommendations to integrate resource theories and grounded theories of resilience (cf. Bryan et al., 2018) we propose that individuals in the sporting context face multiple simultaneous stressors/adversities of varying magnitudes. Sporting resilience is an oscillatory process in response to each stressor and adversity that develops through the individual's response to these adversities. It is not an isolated linear process with a discrete start-middle-end. This conceptualization of resilience as a dynamic process unfolding over time has found empirical support (see Galli and Vealey, 2008; Galli and Gonzalez, 2015; Bryan et al., 2018; Galli and Pagano, 2018; see also Bonanno, 2004, 2012; Bonanno and Diminich, 2013). Initial evidence from resilience research in sport psychology, where studies have included multiple data collection points/longitudinal designs, supports this conceptualization (Secades et al., 2016; Ueno and Suzuki, 2016; Timm et al., 2017; Codonhato et al., 2018; Morgan et al., 2019; Sorkkila et al., 2019). Taken together, they support resilience as a construct to have temporal stability.

Components and outcomes of resilience, however, change over time (Hill et al., 2018a) as the individual is exposed to different environments. The meta model of sporting resilience yields a relatively stable snapshot of the resilience process determined by the individual's interaction-dominant biopsychosocial protective factors. The biopsychosocial filter expands and affords an evidence-basis and testability of the “personal assets” in Fletcher and Sarkar's (2012) definition.

One theoretical advancement is that the individual develops the protective filter comprising biopsychosocial protective factors of resilience and is available when faced with a stressor. Included components of the biopsychosocial protective filter have already received preliminary empirical support (outline in Table 2). The presence or absence of these protective components determines the strength of the protective filter. In an individual case, we can measure objectively each component of this filter through established means (see Table 2). A strong and expansive protective filter grants an individual with available biopsychosocial resources to deploy to overcome an adversity which dilutes the magnitude of the adversity and its effect on the individual during the initial resilience response. For example, if an injured athlete is in a facilitative environment, receives medical and psychological support, has optimism and self-regulatory ability and a strong sense of meaning, the injured athlete finds it easier to engage resiliently against the adversity and adapt positively compared to an injured athlete who does not possess these protective resources (Podlog et al., 2014). Despite a strong protective filter; however, the individual must have a resilient response to the adversity because resilience does not imply an absence of negative pathological consequences post adversity (Southwick et al., 2014).

The protective filter is interaction dominant. It builds through dynamic individual-environment interaction because resilience includes positive adaptations which are determined by culturally/sport specific milestones which are socially constructed (Walsh, 2002; Ungar, 2008; Wagstaff et al., 2016). For example, positive adaptation is different in different sports and signposts unique things to different individuals. Sporting resilience is also environmentally adaptable because the origin of adversity is important, but so is the timing and type of adversity in the phenomenological experience (Sarkar and Fletcher, 2013; Brown et al., 2015). For instance, metacognitive challenge appraisal of the athlete will be different when they are recovering from an injury compared to when they are about to compete in the finals of an elite level global competition. This protective filter is not rigid but malleable and determines an individual's “Homeostatic Resilience” in line with extant conceptualisations that there is a stable level of resilience before perturbations and adversity (Richardson et al., 1990). The components of the protective filter do not have a fixed rank hierarchy but are subjectively evaluated to determine centrality as different stressors affect different athletes in different ways (Thelwell et al., 2007; Sarkar and Fletcher, 2014). Every individual athlete is idiosyncratic. They will not only possess but will rely on particular protective resources in different manners. For example, a sense of meaning and/or passion can be interpreted subjectively differently by two different athletes. The protective filter encompasses the proactive-protective (robust resilience) [i.e., resources which contribute to resilience (Fletcher and Sarkar, 2016)].

So how does the model work? The model showcases the resilience process of the individual at a specific period of linear time. It can trace resilient adaptation to inform the “how-what-where-when” of interventions. We start with adversity, which is percolated through the Biopsychosocial Filter. The stronger the filter is, the weaker the influence of the adversity on the individual. To use a metaphor, the filter acts like a tea strainer. The stronger the filter (resilience protective resources), the more tea leaves (adversity) it filters out. After this, the resilience response of the individual is initiated in one of two trajectories (see Figure 3).

The sporting resilience model reflects aspects of Bonanno and Diminich (2013) model, who noted that resilience has two potential pathways depending on the relevance and magnitude of the adversity. We extend this theorization by stating that the resilience response trajectories are determined by the strength of the protective filter and the response capacity. This process draws parallel from biological immune systems. A strong immune system can either protect the body entirely from illness with no adverse disruption or can engage in a defending process which disrupts internal biological homeostasis, eventually leading to health recovery (Miller and Maner, 2011; Kotas and Medzhitov, 2015) and from protective factors in 5P psychotherapy formulation model. If the protective filter is strong, it filters the adversity to a manageable level, resulting in “minimal impact resilience” (Bonanno and Diminich, 2013, p. 380). As a result, the individual maintains wellbeing and resiliently adapts using existing resources. They only dip slightly below the level of homeostatic resilience and equilibrium functioning (see Figure 3). If the protective filter is weak, however, the adversity is not filtered, and the individual engages in “emergent resilience” (Bonanno and Diminich, 2013, p. 379), and undergoes disruption to the equilibrium performance and shifts from homeostatic resilience.

In the emergent resilience trajectory, there is (1) a disruption of the equilibrium level of functioning that characterizes daily life and routine of the individual; (2) depletion of personal resources and performance because of the intensity of the adversity overwhelming the strength of the protective filter; (3) metacognitive learning *via* self-reflection of the experience and development of existing and new protective resources strengthening the filter; (4) rebound process with newly learned resources and a stronger filter which allows the individual resiliently adapt to the adversity leading to positive adaptation. The resilience process is reactive-integrative (rebound resilience) (Fletcher and Sarkar, 2016).

In time, both trajectories lead to eventual positive adaptation characterized by return to homeostatic resilience and equilibrium functioning. In the sporting context, this is characterized by positive mental health and positive sport-specific performance levels. Sporting resilience is a dialectical and iterative process. Qualitative evidence indicates that it goes beyond a single cycle of reconfiguration and reintegration but involves a positive link between many experiences with adversity and resilient learning from adversity encounters (Fletcher and Sarkar, 2012; Brown et al., 2015). Resilience is a break-build or learning-relearning process that happens with every adversity experience over time.

When the adversity is of a high magnitude, the individual takes trajectory B (i.e., emergent resilience). The individual possesses the appropriate adaptive resources to appraise the diverse range of adversity as a challenge rather than threat post disruption and depletion stages. This results in identification of new possibilities (Day, 2013), *via* metacognitive learning and utilization of resources in a dynamic, interaction-dominant process. This extends the diversifying experience model on creativity and multiculturalism (Gocłowska et al., 2018). This dialectic process is environmentally adapted to suit requirements and is proactive in identifying and reactive in using extant and/or new resources, resulting in a return to positive equilibrium/adaptation. The strength of the protective filter also determines the “resilience apex” (i.e., the limit of resilience response as determined by the strength of the protective filter, much like muscle strength determines lifting capacity). If an acute level adversity is prolonged, the individual's protective factors cannot enable positive adaptation, much like low strength and endurance cannot sustain the physiological load. Where adversity is a high magnitude and prolonged, even with a strong protective filter the individual may engage in emergent resilience trajectory and get trapped in stage II- Depletion, leading to a continuous depletion of resources resulting in a downward negative spiral (Fredrickson, 2001) which eventually crosses the resilience apex resulting in a “critical adaptation failure.”

### Applying the meta-model of sporting resilience

The sporting resilience meta-model has been developed for research and practice, cognizant of the fact that stress and adversity are necessary conditions for resilience (Masten, 2001; Masten and Reed, 2002; Sarkar and Fletcher, 2014; Galli and Gonzalez, 2015; Gonzalez et al., 2018). The protective filter can be expanded as future empirical research cements the existing variables cited and/or discovers new variables which act as protective factors of resilience in sport. Conversely, as future research disproves the evidence supporting a specific component, it can be discounted as a protective component. This provides future research with a clear target of biopsychosocial components to test empirically leading to inclusion or exclusion from the list of protective resources. Using the meta-model will provide the opportunity to assess the interplay of protective factors and capture the dynamicity of the resilience process (Hill et al., 2020).

In applied practice, the model can be used to assess an individual athlete's protective filter, and trajectory of resilient adaptation. Using assessment interviews and formulation, the practitioner can map the protective factors available to the individual at that given moment in time to formulate their protective filter. As a need-analysis and/or a diagnostic guide, this will allow the practitioner to inform interventions and chart potential process-trajectories of resilience. Practitioners who use CBT/REBT in practice can utilize this model as part of their assessment and formulation stage. This model can be used as an initial self-report tool and responses could be then triangulated *via* psychometrics, therapeutic formulation, and observational data (see Table 2). The model can also evaluate the longitudinal duration of interventions and evaluate efficacy. For instance, after the protective filter has been identified, psychotherapy can strengthen prevailing components (i.e., highlight the self-identification and interaction between components to increase resilience *via* building biopsychosocial resources) (Mandrekar and Gupta, 2022).

Results of this systematic review indicate that interventions to build resilience is aligned to stress inoculation theorisation as stress experience builds mastery and improves resilience *via* reintegration (Flach, 1988, 1998; Galli and Vealey, 2008; Fletcher and Sarkar, 2016; Kegelaers et al., 2019). There are also suggestions that moderate cumulative lifetime adversity is associated with more positive responses to subsequently encountered stressors (Moore et al., 2018). Relevant to this is establishing an environment which balances challenge and support (Fletcher and Sarkar, 2016) which leads to acceptance of the adversity and seeking meaning/comprehension of adversity (Howells and Fletcher, 2015). Following this, there is a consequent positive reframing of negative experience and derogation of adversity related experiences allow athletes to perceive adversities in a different light to develop a positive bias in the future. Considering these recommendations, we advocate using the systematic self-reflection model of resilience (Crane et al., 2019) for resilience intervention in sport to improve the metacognitive, perceived/tangible social support, self-insight and self-regulation components of sporting resilience by enhancing the strength of the protective filter. Caution must be taken to be mindful of the magnitude of stressors provided and tailor it to the strength of the individual's protective filter to prevent the response exceeding the resilience apex and resulting in critical adaptation failure. We recommend using the “adversity exposure matrix” (Bryan et al., 2019, p. 80) which can be used with the meta-model of sporting resilience to determine the process-trajectory of sporting resilience in specific idiosyncratic cases by practitioners.

### Implication and directions for future research

A major significance of this review is the method applied. We showcase and extend a template of theoretical advancement in psychological science, building upon the work of Fredrickson (2001) and Jones et al. (2009). We start from search and review of evidence (theoretical and empirical) already present, describe and evaluate the evidence, integrate empirically validated components into an explanatory theoretical frame. Because of the rigorous systematic review process and scientist-practitioner focus of the theory and definition, using this definition for future research rather than proposing novel operationalization's constitutes better use of resources and will develop scientific knowledge in positive psychology.

This study has undertaken a systematic synthesis of resilience literature to offer an evidence based operational definition and meta-model of sporting resilience. The definition of sporting resilience provided can be used by researchers to consolidate the operationalization of resilience research in sport. This will allow replicability of findings and greater consensus in evidence. For example, the frequency counts of supporting evidence given in Table 2 can be used by researchers to better understand the gap in evidence. The sporting resilience meta-model offers a theoretical model explaining the process-trajectory of how resilience unfolds and for applied practice assessment and interventions. Specifically, the model can be used in youth sport to develop sporting resilience profiles of young athletes and linking it to talent development for elite performance, injury rehabilitation, and performance slumps. At this stage, the model is suited to guiding practitioners rather than providing a prescriptive blueprint because theory is an ongoing process rather than an established fact (Morse, 1997). Future research needs to add to the sporting resilience meta-model to confirm/refute components as the evidence base grows. The conceptual advances of this model have been validated preliminarily by the existing data this systematic review analyzed and by a grounded theory investigation. It can be tested in other samples in research projects.

This systematic review included longitudinal studies and evidence synthesized from findings, providing initial support for the predictive stability of resilience as a construct while highlighting its constancy. Findings support the notion that resilience is an exclusive psychological construct and does not risk construct redundancy with constructs such as hardiness and/or grit (Martin et al., 2015). Therefore, directions entreated by Galli and Gonzalez (2015) and Bryan et al. (2019) have initial evidence to support resilience to be a predictive, moderately stable state-like process determined *via* person-environment interactions. Our review supports the findings of Bryan et al. (2019) in stating that most measures of resilience view it as a trait and there is a pressing need for a sport specific measure. We recommend future research to use the operationalisation of sporting resilience as the foundation for psychometric development (Hinkin, 1995).

## Conclusion

From its infancy in the early 2000s to the robust growth in the last decade, the science of resilience is growing. Resilience is being heralded by the lingua franca of psychology research and applied practice. Considering the massive rupture in the continuity and normalcy of sports worldwide that is expected to follow in the aftermath of COVID-19, resilience has never been more important (Gupta and McCarthy, 2021).

Sporting resilience and its meta-model proposed in this systematic review is rooted in Vealey (2006) prompt to “examine the box” (p. 129) of a paradigm to maintain the much-needed wonder in investigative inquiry. This systematic review provides a comprehensive overview of the existing epistemological base of resilience research in sports psychology whilst striving to push its ontological box. While results of this systematic review indicate that resilience research has permeated to Non-WEIRD (western, educated, industrialized, rich, democratic) contexts (Henrich et al., 2010), research in sporting resilience should strive to be inclusive of cross-cultural theory and praxis, because sport is transcultural (Gupta, 2022; Gupta and Divekar, 2022). The operationalisation of Sporting Resilience and the meta model allows a more systematic empirical examination of the construct in sport psychology.

## Data availability statement

The original contributions presented in the study are included in the article/Supplementary material, further inquiries can be directed to the corresponding author/s.

## Author contributions

SG: research ideation, design, data collection, data analysis, conceptual model, definitional advancement, and manuscript preparation. PM: research methodology, data analysis, and manuscript preparation. All authors contributed to the article and approved the submitted version.
