# Supplementary material for: The sporting resilience model: A systematic review of resilience in sport performers
Source: Front Psychol. 2022 Dec 21;13:1003053. doi: 10.3389/fpsyg.2022.1003053 (PMC9811683; doi:10.3389/fpsyg.2022.1003053)
Supplement: Supplementary file 1 [file Data_Sheet_1.docx]

**Appendix A**

**Adapted Quality/Risk of Bias Appraisal Tools**

**MMAT-Adapted (**Pluye & Hong, 2014)

1. **Qualitative Criteria**

**C1-** Is the sample source of the qualitative data relevant to address the research question?

**C2-** Does the study provide description of data collection, data checks and analysis?

**C3-** Is the analysis process relevant to address the research question?

**C4-** Does the study provide considerations how findings relate to the context of the study?

**C5-** Is there a discussion of researcher reflexivity in the findings?

1. **Quantitative Data**

**C1-** Is the sampling strategy relevant to address the research question?

**C2-** Is the study sample representative of the population under study? (specifically, age, sex, competitive level, type of sport)

**C3-** Are the measurements used appropriate and reliable? (justification of measurement selection)

**C4-** Does the study adequately control confounding variables?

1. **Mixed Method Criteria**

**C1-** Are the measurements used appropriate and reliable?

**C2-**Is the study sample representative of the population under study? (specifically, age, sex, competitive level, type of sport)

**C3-**Is the mixed-methods research design relevant to address the qualitative and quantitative research questions, or the qualitative and quantitative aspects of the mixed-methods question?

**C4-** Is the integration of qualitative and quantitative data relevant to address the research questions?

**C5-** Is appropriate consideration given to the limitations associated with this integration, in a triangulation design?

**JBI Systematic Review Checklist (**Joanna Briggs Institute, 2017)

**C1-** Is the review question clearly and explicitly stated?

**C2-** Is the inclusion criteria appropriate for the review question?

**C3-** Is the search strategy appropriate/ are the scope of papers included comprehensive?

**C4-** Are the sources and resources utilised to search for the studies adequate?

**C5-** Is the critical appraisal conducted by multiple reviewers independently/ is critical appraisal conducted and justified?

**C6-** Are there methods to minimize errors in data extraction/do authors provide an unbiased review of existing literature?

**C7-** Is the methods used to synthesise literature appropriate/ does the synthesis and presentation of evidence have clarity?

**C8-** Is the likelihood of publication bias assessed?

**C9-** Is the recommendations listed supported by reported data?

**C10-** Are they specific directives for future research?

**JBI Appraisal Checklist adapted for Conceptual Papers- Adapted (**Joanna Briggs Institute, 2017)

**C1-** Is the source of the publication clearly identifiable?

**C2-** Is the aim clearly defined and the central focus of the study?

**C3-** Is the stated position the result of analytical, reasoned process supported by logic?

**C4-** Is there reference to extant literature?

**C5-** Is congruence/incongruence with extant literature/conceptual frameworks logically supported or defended?

**Appendix B**

**Quality appraisal of included studies (Quantitative Studies)**

| Study | | Authors | Quantitative Criteria | | | | Quality Score | Notes | Theoretical/Conceptual Framework | Sample | Measures |
| --- | --- | --- | --- | --- | --- | --- | --- | --- | --- | --- | --- |
|  | | | C1 | C2 | C3 | C4 |  |  |  |  |  |
| Explanatory style as a mechanism of disappointing athletic performance | Seligman, M.E., Nolen-Hoeksema, S., Thornton, N., & Thornton, K.M. (1990) | | Y | Y | Y | N | 75 | Resilience not a variable studied, but rather utilised as a discussion point for results and linked to explanatory style and athletic performance | No theoretical model used (in defense, study was conducted in 1990 when there were no theoretical models of resilience) | International level  American varsity  swimmer (*n*= 48) | Attributional Style questionnaire and objective measures of sport-specific performance |
| Conjunctive Moderator Variables in Vulnerability and Resiliency Research: Life Stress, Social Support and Coping Skills, and Adolescent Sport Injuries | Smith, R.E., Smoll, F.L., & Ptacek, J.T. (1990) | | Y | Y | N | N | 50 | Coping related measurement of resilience used. Highly important study since it outlined conjunctive pattern of moderator protective variables and how they maximise outcome effect of resilience | No theoretical model used (in defense, study was conducted in 1990 when there were no theoretical models of resilience) | Varsity athletes  in high school (*n*=451) | No resilience measurement used. Studied what is now considered to be protective factors of resilience via Athletic Coping skills inventory (Smith et al., 1988). |
| Explanatory style and resilience after sports failure | Martin-Krumm, C.P., Sarrazin, P.G., Peterson, C., Famosed, J (2003) | | N | Y | N | N | 25 | Participants were general school children not athletes. Entire study mentions resilience once, in the title | No theoretical model of resilience used | School basketball  players (*n*=62) | Physical basketball dribbling |
| Bouncing Back: The Role Of Coping Style, Social Support And Self-Concept In Resilience of Sport Performance | Mummery, W.K., Schofield, G., & Perry, C. (2004) | | Y | Y | N | N | 50 | Naturalistic study but no resilience scale used. Grouping as participants not theoretically motivated. | No theoretical model; operationalisation of resilience borrowed from general psychological theory | National level  Australian swimmers  in age-group (*n*=272) | Athletic Coping skills inventory (Smith et al., 1995) |
| Stress-Resilience, Illness, and Coping: A Person-Focused Investigation of Young Women Athletes | Yi J.P., Smith R. E., & Vitaliano P. P. (2004) | | Y | Y | N | Y | 75 | Unusual operationalisation of resilience, focusing more on physiological resilience rather than psychological resilience | No theoretical model of resilience used. Resilience operationalised in terms of high life stress and low illness. | Female high  school athletes  (*n*=404) | Revised Ways of Coping Checklist (Vitaliano et al, 1985) and Life Stress-Adolescent Perceived Events Scale (Compas, Davis, Forsythe & Wagner, 1987; Smith et al., 1990) |
| Relations of resilience and hardiness with sport achievement and mental health in a sample of athletes | Nezhad, M.A., & Besharat, M.A. (2010) | | Y | Y | N | N | 50 | Poor operationalisation of contructs of resilience and hardiness. Data collection profesure and ethical framework not provided. | No theoretical model of resilience used | Club level  athletes  (*n=*149) | Connor Dacidson Resilience Scale (2003)- 25 item version |
| Relation of resilience whit sport achievement and mental health in a sample of athletes | Hosseini, S.A., & Besharat, M.A. (2010) | | Y | Y | N | N | 50 | Method and data collection rpocedure not mentioned adeqautely. Poorly cited introduction and no mention of ethical considerations | No theoretical model of resilience used | Club level  Volunteer  athletes  (*n*=139) | Connor Dacidson Resilience Scale (2003)- 25 item version |
| The Connor-Davidson Resilience Scale (CD-RISC): Dimensionality and age-related measurement invariance with Australian cricketers | Gucciardi, D.F., Jackson, B., Coulter, T.J., & Mallett, C.J. (2011) | | Y | Y | Y | Y | 100 | CDRISC-10 has adeqaute model fitness and inavariance across playing experience, but has questionable internal reliability | N/A | Adolescent Australian  cricketers (*n=*191)  and State/ national  athletes (*n*=321) | Connor Davidson Resilience Scale (2003), 10-item, 22-item and 25-item |
| Resilience scale for Athletes | Subhan, S., & Ijaz, T. (2012) | | N | N | N | N | 0 | Non-WEIRD (Heinrich et al., 2010) sample scale. However, Qualitative component not adequate for item construction, poor psychometrics, no concurrent validity check with existing scales, comprehension level doubts since instructions in Urdu but the scale is in English, poor sampling. | No theoretical model of resilience used for scale development | College athletes  (*n*=150) | Resilience Scale for Athletes (RSA)- study developed |
| Resilience of athletes with physical disabilities: A cross-sectional study | Cardoso, F.L., & Sacomori, C. (2014) | | N | Y | N | N | 25 | Poor reporting of data (sample size different in analysis and in reported statistics which reduces fidelity of results), no details of sampling procedure provided. However, excellent aim and direction for future research for resilience in sporting context. | No operational definition or theoretical framework used | Brazilian athletes  with physical  disabilities (*n*=208),  disabilities classified  as congenital  or acquired | Wagnild and Young (1993) Resilience Scale |
| The role of the resilience in coping with stress in sports | Bejan, R., & Tonita, F. (2014) | | N | N | N | N | 0 | Study merely reported score of resilience scale, no comparison, or descriptive/inferential analysis measures. Despite noting coping with stress in the title, no analysis has been conducted of the same. | No operational definition or theoretical framework used | Teenage  tennis  players (*n*=4) | Wagnild and Young (1993) Resilience Scale- adapted and translated |
| Impact of coping strategies on resilience of elite beach volleyball athletes | Belem, I.C., Caruzzo, N.M., Roberto, J., do Nascimento Jr, A., Vieira, J.L., & Vieira, L.F. (2014) | | Y | Y | Y | Y | 100 | Cross-sectional study, data collected at technical conference not in sport environment | Richardson et al., (1990) Resiliency Model | Brasil Beach  volleyball  athletes (*n*=48) | Connor Davidson Resilience Scale (2003), 10-item |
| Gritty, Hardy, and Resilient: Predictors of Sport Engagement and Life Satisfaction in Wheelchair Basketball Players | Martin, J.J., Byrd, B., Watts, L.M., Dent, M. (2015) | | Y | N | Y | Y | 75 | Study is an in-depth analysis of the variables and provides important evidence against construct redundancy. However, no females and limted sample limit generalizability. There is no focus on psychosocial factors. This is important because data was collected during tournament potentially causing bias | No Guiding theoretical model of resilience | 75 (74M 1F) | CDRISC-10 (Connor & Davidson, 2003) |
| The Cortisol Awakening Response and Resilience in Elite Swimmers | Meggs, J., Golby, J., Mallett, C. J., Gucciardi, D. F., Polman, R. C. J. (2016) | | Y | Y | Y | Y | 100 | Psychometric and physiological measures of resilience, however, there may be other mediating variables such as metacognitive interpretations of emotions and consequent physiological responses. | Grounded theory of psychological resilience (Fletcher & Sarkar, 2012) to discuss findings | International/national  level English  and Australian  swimmers (*n*=41) | Academic Resilience Scale (Martin & Marsh,2006)., adapted for the sports context |
| Motivational climate, resilience, and burnout in youth sport | Vitali, F., Bortoli, L., Bertinato, L., Robazza, C., & Schena, F. (2015). | | Y | Y | Y | Y | 100 | No control for demographic/social factors influencing resilience. Provides cross-sectional evidence for mastery climate fostering resilience | Achievement Goal Theory (Nicholls, 1984) | Adolescent basketball  athletes (*n*=87) | Connor Davidson Resilience Scale (2003), 10-item |
| Validity and reliability of the Connor-Davidson Resilience Scale (CD-RISC) in competitive sport | Gonzalez, S. P., Moore, E. W. G., Newton, M., & Galli, N. A. (2016) | | Y | Y | Y | Y | 100 | CDRISC-10 was the best fitting model and has no measurement differences in relation to gender. However, long distance runners sample denotes that it is a sport where experience can influence metacognition component of resilience, which may limit generalizability to other sports. | N/A | Post-collegiate  long-distance  runners  (*n*=405) | Connor Davidson Resilience Scale (2003), 10-item, unidimensional version and five factor version |
| Self-reflection and Self-insight Predict Resilience and Stress in Competitive Tennis | Cowden, R. G., & Meyer-Weitz, A. (2016). | | Y | Y | Y | Y | 100 | It cannot be determined whether insight/reflective components operate in competitive situations since data was collected in non-competitive period | No theoretical model of resilience used | Competitive  tennis athletes  (*n=*333) | Resilience Scale for Adults (Friborg et al.,2005) |
| Longitudinal study on the relationship between resilience and burnout among Japanese athletes | Ueno, Y., & Suzuki, T. (2016) | | Y | Y | Y | Y | 100 | Longitudinal study collecting data in three period across the year. However, being in university, academic stressors and response may have played a role in the resilience process. | No operational definition or theoretical framework used | Japanese university  athletes who  compete at  national/regional  levels (*n*=63) | Psychological Resilience Scale for University Athletes (Ueno & Shimizu, 2012) |
| Associations of motivation, self-concept and resilience with the competitive level of Chilean judokas | Zurita-Ortega, F., Muros-Molina, J. J., Rodriguez-Fernandez, S., Zafra-Santos, E. O., Knox, E., & Castro-Sanchez, M. (2016). | | Y | Y | Y | Y | 100 | Operationalization of resilience as an endogenous variable | No theoretical model of resilience used | Chilean judokas  of the  professional/  semi-professional /  amateur level  (*n=*148) | Connor Dacidson Resilience Scale (2003)- 25 item version |
| Relationship Between Resilience and Coping Strategies in Competitive Sport | Secades, X. G., Molinero, O., Salguero, A., Barquín, R. R., de la Vega, R., & Márquez, S. (2016) | | Y | Y | Y | Y | 100 | Data collected at two separate points; the end of last competitive mesocycle and post important competition. | Grounded theory of psychological resilience (Fletcher & Sarkar, 2012); Conceptual Model of | Spanish athletes  of team  and individual  sports (*n*=235) | Spanish version of Wagnild and Young (1993) Resilience Scale |
| Interaction of athletes' resilience and coaches' social support on the stress-burnout relationship: A conjunctive moderation perspective | Lu, F. J., Lee, W. P., Chang, Y. K., Chou, C. C., Hsu, Y. W., Lin, J. H., & Gill, D. L. (2016). | | Y | Y | N | Y | 75 | Cross-sectional study in an Non-WEIRD context | Grounded theory of psychological resilience (Fletcher & Sarkar, 2012) to discuss findings | Individual  and team sports-  student athletes  (*n*=218) | Connor Davidson Resilience Scale (2003)- 2 item version |
| Evaluating the Level of Exercise Dependence and Psychological Resilience of Athletes from Different Branches | Bingol, E., & Bayansalduz, M., (2016) | | N | Y | N | N | 25 | No rigour, study presents a descriptive report, poor rationale and no clear aim and ethical consideration in study conduction reported | No Guiding theoretical model of resilience | 777 (313F 464M) participants  in sport for 5 years, aged 22-25 years | Ego Resiliency Scale (Block & Kremen, 1996) |
| Athletes Engagement, Resilience, and Rate of Perceived Exertion on Portuguese National- and International-Level Wrestlers | Pedro, G.D., S. (2016) | | Y | N | Y | Y | 75 | Small sample only limited to Portuguese wrestlers, too few females limiting ecological generalizability. Did not discuss how/why engagement is related to resilience. | No Guiding theoretical model of resilience | 20 International Portuguese  Wrestlers (17M 3F) | Resilience Scale 13-A (Wagnild & Young, 1993)- adapted to Portuguese |
| Evaluation relationship between resilience and physical activity levels of national sports | Metin, S. A. H. I. N., SAGIRKAYAAli, L. O. K., Kerime, B. A. D. E. M. L. I., Hicran, T. K., & Sefa, L. O. K. (2017) | | Y | N | N | Y | 50 |  | No theoretical model of resilience used | Turkish taekwondo  athletes (*n*=165) | Resilience Scale for Adults (Friborg et al.,2005) |
| Resilience in Adapted Paddle coaches | Ruiz-Barquín, R., de la Vega-Marcos, R., De la Rocha, M., & Ortín-Montero, F. J. (2017). | | Y | N | Y | Y | 75 |  | No theoretical model of resilience used | Padle trainers  (*n*=118) | Wagnild and Young (1993) Resilience Scale |
| Resilience and recovery-stress in competitive athletes | García-Secades, X., Molinero, O. R., Ruiz-Barquín, R., Salguero, A., De la Vega, R., & Márquez, S. (2017). | | Y | Y | Y | Y | 100 | Data collected at two separate points; the end of last competitive mesocycle and post important competition. Recovery-stress questionnaire is utilised to track difference between collection points but specificity of recovery/stressor not noted | No theoretical model of resilience used | International/  national/  regional  levels  (*n*=235) | Spanish version of Wagnild and Young (1993) Resilience Scale |
| Formation of resilience in Japanese athletes: Relevance to personality traits and day-to-day resilience | Ueno, Y., & Oshio, A. (2017) | | Y | Y | Y | N | 75 | Operationalisation of resilience has no backing of literature, and no control for antecedent confounding variables. Athlete resilience as a dependent variable could have been directly affected by personality. | No theoretical model of resilience used | Japanese international/  national/  regional  athletes  (*n*=165) | Psychological Resilience Scale for University Athletes (Ueno & Shimizu, 2012) |
| Development and Validation of the Characteristics of Resilience in Sports Teams Inventory | Decroos, S., Lines, R. L., Morgan, P. B., Fletcher, D., Sarkar, M., Fransen, K., ... & Vande Broek, G. (2017). | | Y | Y | Y | Y | 100 | Team resilience scale has good reliability, structural invariance, with retest as well. Suppoerts socioecological perspective of resilience in sports teams. | N/A | Belgium and  UK athletes  from  team sports  (*n*=1225) | Scale developed in study, Study 1= Cognitive interviews with academics for item validation; Study II/III= modified scales post ICM-CFA |
| What coping strategies are used for athletes of MMA more Resilience to Stress? | Belem, I. C., Santos, V. A. P. D., Caruzzo, N. M., Rigoni, P. A. G., Both, J., & Vieira, J. L. L. (2017). | | Y | Y | Y | N | 75 | Study conducted in a sport with modeality differences. However, grouping of pre-competition and general prep period not analysed in terms of temporl differences which could be a counfounding variable influence fidelity of findings. | Galli and Vealey (2008) Conceptual Model of Resilience; Richardson et al., (1990) Resiliency Model | Brazilian National/  international Male  MMA fighters  (*n*=62) | Connor Davidson Resilience Scale (2003), 10-item, validated for Brazilian context |
| The Effect of Unity in Sport Teams on Athletes' Mental Health: Investigating the Mediating Role of Resilience | Yamada, K., Kawata, Y., Kamimura, A., & Hirosawa, M. (2017). | | Y | Y | Y | Y | 100 | Methodological contribution for integration of socioecological and mental health components. | No operational definition or theoretical framework used | Japanese university  athletes who compete international/  national/  regional level  (*n*=626) | Psychological Resilience Scale for University Athletes (Ueno & Shimizu, 2012) |
| Psychological Resilience’s Moderation of the Relationship Between the Frequency of Organizational Stressors and Burnout in Athletes and Coaches | Wagstaff, C., Hings, R., Larner, R., & Fletcher, D. (2018). | | Y | Y | Y | Y | 100 | Studied sociocultural- Environmental influence of resilience, but causality is not establish since it is a cross-sectional study. Variablity of organisational stressors for types not noted. | Grounded theory of psychological resilience (Fletcher & Sarkar, 2012); Conceptual Model of | Study 1=Team/  individual sport athletes from  all levels of competition(*n*=1372),.  Study 2= Coaches at all  levels of competition  from team and  individual sports (*n*=91) | Connor Davidson Resilience Scale (2003), 10-item |
| Confirmatory factor analysis of the Brief Resilience Scale for Brazilian athletes | Neves, A. N., Barbosa, F. P., Da Silva, M. P., Brandão, M. R., & Zanetti, M. C. (2018) | | Y | Y | Y | Y | 100 | Findings suggest covariance error for 4 items of the scale suggesting similarity of content or social desirability | N/A | Beginner/  skilled/national/  international  athletes (*n*=330) | Brief Resilience Scale (Smith et al, 2008), Brazilian Portuguese adaptation |
| The Prevalence of Depressive and Anxiety Symptoms in Student-Athletes and the Relationship with Resilience and Help-Seeking Behavior | Drew, B., & Matthews, J. (2019) | | Y | Y | Y | Y | 100 | Comprehensive and well-designed study. However, clinical interviews and observations could have been better suited as an evidence source. Study could also have provided more discussions on the role and specific mechanisms of resilience as protective factor in depressive and anxiety symptoms | Grounded Theory of Psychological Resilience (Fletcher & Sarkar, 2012) | CDRISC-10 (Connor & Davidson, 2003) | Comprehensive and well-designed study. However, clinical interviews and observations could have been better suited as an evidence source. Study could also have provided more discussions on the role and specific mechanisms of resilience as protective factor in depressive and anxiety symptoms |
| IMPACT OF RESILIENCE ON STRESS AND RECOVERY IN ATHLETES | Codonhato, R., Vissoci, J. R. N., Nascimento Junior, J. R. A. D., Mizoguchi, M. V., & Fiorese, L. (2018) | | Y | Y | N | Y | 75 | Sample selection strategy not mentioned. Recovery stress questionnair has questionable reliability, and details/magnitude of injury not mentioned | Galli and Vealey (2008) Conceptual Model of Resilience | Top 3 placers in  2012 Parana  Open Games  Finals (*n*=150) | Connor Davidson Resilience Scale (2003), 10-item |
| Examining the process of psychological resilience in sport: performance, cortisol and emotional responses to stress and adversity in a field experimental setting | Gonzalez, S. P., Newton, M., Hannon, J., Smith, T. W., & Detling, N. (2018) | | Y | Y | Y | Y | 100 | In line with recommendations from literature of combining physiological measures with self-report psychological indices in a field experimental setting. Rigourous method, fidelity and manipulation checks conducted. Blinding for group assignment | Grounded theory of psychological resilience (Fletcher & Sarkar, 2012); Conceptual Model of | Collegiate  lacrosse players  (*n*= 54;  High Resilience= 18,  Low Resilience = 18; Control Group= 17). | CDRISC-10 (Connor & Davidson, 2003); Salivatory Cortisol measure for physiological index. |
| Relationship of resilience, anxiety and injuries in footballers: Structural equations analysis | Zurita-Ortega, F., Chacón-Cuberos, R., Cofre-Bolados, C., Knox, E., & Muros, J. J. (2018). | | Y | Y | Y | Y | 100 | Findings is limited to male only and cannot be generalized | No theoretical model of resilience used | Male footballers (*n*=185) | Connor Davidson Resilience Scale (2003), 10-item |
| Grit, self-regulation and resilience among college football players: A pilot study | Gupta, S., & Sudhesh, N. T. (2019) | | Y | N | Y | Y | 75 | Results illustrated a potential relationship of resilience with self-regulation in team sports but not with Grit which is a more individual level trait. | Grounded Theory of Psychological Resilience (Fletcher & Sarkar, 2012) | College footballers  of 18-24 years of age  (*n=* 32,  16M;  16F). | BU Resilience Scale (Annalakshmi, 2009) |
| Levels of Physical Activity Are Associated With the Motivational Climate and Resilience in University Students of Physical Education From Andalucía: An Explanatory Model | Chacón-Cuberos, R., Castro-Sánchez, M., Pérez-Turpin, J. A., Olmedo-Moreno, E. M., & Ortega, F. Z. (2019) | | Y | Y | Y | N | 75 | Sample are physical education students at University and may have been ex-post predisposed to sport and physical activity | No theoretical  model of resilience  used | Physical  Education  students  (*n*=775) | Connor Dacidson Resilience Scale (2003)- 25 item version, validated into Spanish by Olmo et al., (2017) |
| The role of resilience in student‐athletes' sport and school burnout and dropout: A longitudinal person‐oriented study | Sorkkila, M., Tolvanen, A., Aunola, K., & Ryba, T. V. (2019). | | Y | Y | Y | Y | 100 | Data collected at four separate points in an ongoing longitudinal study, but resilience was not longitudinally tracked. Study is in Finland which is a differently structured societal/school/sport environment | No theoretical  model of resilience used | School student  athletes (*n*=491) | Brief Resilience Scale (Smith et al, 2008) |
| The influence of self-regulated learning and coping styles on psychological resilience in sports | Kegelaers, J., Wylleman, P., & Tas, M. (2019). | | Y | Y | Y | Y | 100 | Study available in Poster format. Highlighted role of belief in addition of specific metacognitive or behaviour strategy as influencing resilience response. | Grounded theory of psychological resilience (Fletcher & Sarkar, 2012); Conceptual Model of Resilience (Galli & Vealey, 2008) | Regional/  National/  International athletes (*n*=235),  team and  individual sports | Brief Resilience Scale (Smith et al, 2008) |
| Investigation of Elite Athlete's Psychological Resilience | Ozdemir, N. (2019) | | Y | N | N | N | 50 | Findings are a psychometric report of resilience in sample. No rigour. Discussion incorrectly generalised. Insufficient demographic details. Provides a Flow-Chart Process model of Psych Resilience in Athletes but not substantiated by any evidence. | Friborg et al., (2005); Richardson et al., (1990) | *n=*147 (79M 68F) | Resilience Scale for Adults (Friborg et al.,2005) |
| Effect of Motivation on the Resilience and Anxiety of the Athlete | Trigueros, R.; Aguilar-Parra, J.M.; Álvarez, J.F.; Cangas, A.J.; López-Liria, R.(2020a) | | Y | N | Y | Y | 75 | Comprehensive analysis and hypothesis testing. However, sampling is insufficient to label as athletes when only volleyball players were included | SDT (Ryan & Deci, 2000)/ No guiding resilience theory | 276 (147M 129F), Volleyball players | Resilience Scale in the Sporting Context (Trigueros et al., 2017) |
| Exploring the role of Resilience and Basic Psychological Needs and Antecedents of Enjoyment and Boredom in Female Sports | Gonzalez, L., Castillo, I., & Balaguer, I. (2019) | | Y | Y | Y | Y | 100 | Relevant findings and discussion, solid theoretical base, findings relevant for female sporting environment | SDT/ No guiding resilience theory | 661 Females (Basketball n=313) (Football n=348) all levels of sport | Spanish version of Wagnild and Young (1993) Resilience Scale |
| Psychological Resilience and Violence Tendency Levels of High School Adolescents Who Doing Team and Individual Sports | Karademir, T., & Gencay, A., O. (2020) | | N | Y | N | N | 25 | Non-WEIRD(Heinrich et al., 2009) sample. However, there is no clear rationale for choice of variables, poor rigour in design and analysis and insufficient discussion of questionable findings. | No Guiding theoretical model of resilience | 225 (121M 104F) 14-18 years, from Football, Basketball, Volleyball, Boxing, Tennis, Athletics, Taekwondo | Child and Youth Resilience Measure CYRM-28 (Ungar & Liebenberg, 2011) |
| Emotion, Psychological Well-Being and Their Influence on Resilience. A Study with Semi-Professional Athletes | Trigueros, R.; Aguilar-Parra, J.M.; Álvarez, J.F., Gonzalez-Bernal., J., J., & López-Liria, R.(2019) | | Y | Y | Y | Y | 100 | Robust model with good size and generalizability. Findings provide preliminary evidence on how the mechanisms through which coach-athlete relationships contribute to sporting resilience in athletes | No Guiding theoretical model of resilience | 547 Semi-Pro Athletes (289M 258F), 16-19 years, multiple sports | Resilience Scale in the Sports Context- adapted from Portugese (Vigário, I., Serpa, S., & Rosado, A., 2009) |
| The Influence of the Trainer’s Social Behaviors on the Resilience, Anxiety, Stress, Depression and Eating Habits of Athletes | Trigueros, R., Mercader, I., Gonzalez-Bernal., J., J.,Aguilar-Parra, J.M., Gonzalez-Santos, J., Navarro-Gomez, N., & Soto-Camara, R., (2020b) | | Y | Y | Y | Y | 100 | Comprehensive analysis, excellent rigour and good discussion. However, specificity of trainer's role is not mentioned i.e. coach/SE etc). There is also a possibility of social desirability bias in the Anxiety, Stress and eating habit psychometrics used. | No Guiding theoretical model of resilience | 1547 athletes (49.97% male),  127 Trainers, from different  Sports- Football, Basketball, Volleyball and Handball | Resilience Scale in the Sports Context- adapted from Portuguese (Vigário, I., Serpa, S., & Rosado, A., 2009) |
| Nonergodicity in Protective Factors of Resilience in Athletes | Hill, Y., Meijer, R. R., Van Yperen, N.W., Michelakis, G., Barisch, S., &den Hartigh, R.J., (2020) | | Y | Y | Y | Y | 100 | Novel study which provides an important preliminary evidence against group level resilience intervention efficacy testing. Casts doubt on the voracity of group level resilience psychometric testing of interventions in past research | Dynamical Systems Model of Resilience  (Hill et al., 2018a) | 62 (21M 41F) | Brief Resilience Scale (Smith et al, 2008), Brazilian Portuguese adaptation |

**Appendix B:** Continued (Qualitative Studies)

| Study | Authors | Qualitative Criteria | | | | | Quality Score | Notes | Theoretical/Conceptual Framework | Sample | Measures |
| --- | --- | --- | --- | --- | --- | --- | --- | --- | --- | --- | --- |
|  | | C1 | C2 | C3 | C4 | C5 |  |  |  |  |  |
| Toward a Grounded Theory of the Psychosocial Competencies and Environmental Conditions Associated with Soccer Success | Holt, N.L., & Dunn, J.G. (2004) | Y | Y | Y | Y | Y | 100 | No operational definition/theoretical conceptualisation of resilience used. However, study published in 2004 has significance in its role of guiding resilience research in sports psychology. | No guiding theoretical framework | Youth football players (*n=*34_ and professional full time coaches (*n=* 6) | Semi-structured interview (schedule provided) |
| “Bouncing Back” From Adversity: Athletes’ Experiences of Resilience | Galli, N. & Vealey, R.S. (2008) | Y | Y | Y | Y | Y | 100 | First model of psychological resilience in sports psychology. Critiques for overreliance on Richardson et al (19902) resiliency model | Richardson et al., (1990) Resiliency Model | Retired professional athletes (*n=10)* | Semi-structured interview |
| “Give It Everything You Got”: Resilience for Young Males Through Sport | Hall, N. (2011) | Y | Y | Y | Y | Y | 100 | Unique area of study, however generalizations are limited due to sample specificity. | Salutongenic model (MacDonald, 2006) | Male athletes of ages 16-25 years from Western Sydney (*n*=14) | Semi-structured interviews for narrative style data |
| A grounded theory of psychological resilience in Olympic champions | Fletcher, D. & Sarkar, M. (2012) | Y | Y | Y | Y | Y | 100 | Widely used model of psychological resilience in sports | No guiding theoretical framework | Olympic champions (*n=*12) | Life story interviews |
| Defining and Characterizing Team Resilience in Elite Sport | Morgan, P.B., Fletcher, D. & Sarkar, M. (2013) | Y | Y | Y | Y | Y | 100 | Operationalised team resilience as a group level concept | Formulated a theory of team resilience via defining it and outlining characteristic features | International level, competitive elite team sport athletes (*n*=31) | Homogenous focus groups of same team members of 5-8 participants, with semi-structured interview guide |
| Resilience in Competitive Athletes With Spinal Cord Injury: The Role of Sport Participation | Machida, M., Irwin, B., & Feltz, D. (2013) | Y | Y | Y | Y | Y | 100 | Outlined a model of resilience post traumatic injury | Richardson et al., (1990) Resiliency Model | Wheelchair rugby athletes (*n*=12) | Self-structured interview |
| Resilience in Youth Sport: A Qualitative Investigation of Gymnastics Coach and Athlete Perceptions | White, R., & Bennie, A. (2015) | Y | Y | Y | Y | Y | 100 | Provided evidence that a positive sports environment facilitates resilience in youth sports contexts | Challenge Model of Resilience (Ferguson & Zimmerman, 2005) | Australian female gymnasts (*n*=22) and Gymnastic Coaches (*n*=7) | Semi-structured interviewing and Grounded Theory |
| In the face of adversity: Resiliency in winter sport athletes | Brown, H.E., Lafferty, M.E., & Triggs, C. (2015) | Y | Y | Y | Y | Y | 100 | Although results are highly sports specific, the study provides illumination on the process of resilience not its outcomes | Galli and Vealey (2008) Conceptual Model of Resilience and Multidimensional conceptualisation of resilience (Lepore & Revenson, 2006) | Elite level Winter sports athletes (*n*=7) | Semi-structured interviews based on Galli and Vealey (2008) |
| Resilience and Growth in Marathon Runners in the Aftermath of the 2013 Boston Marathon Bombings | Timm, K., Kamphoff, C., Galli, N., & Gonzalez, S.P. (2017) | Y | Y | Y | Y | Y | 100 | Exploration of a naturalistic highly traumatic event and the its impact on resilience via sport. Retrospective exploration of how resilience unfolded over a period of time | Galli and Vealey (2008) Conceptual Model of Resilience | Competitors of 2013 Boston Marathon (*n*=16) | Semi-structured interview with interview guide based on Galli & Vealey (2008). |
| Exploring the Coach’s Role in Fostering Resilience in Elite Athletes | Kegelaers, J. & Wylleman, P. (2018) | Y | Y | Y | Y | Y | 100 | Experiential Interview Scheme is a methodological advancement and results indicate that coaches play a major role in developing positive environments fostering resilience. However, study is non-inclusive and not ecologically valid since no females were included | Process Conceptualization of Resilience (Luthar et al., 2000) | Male athletes (*n*=4), male coaches (*n*=4), male and female sports psychologists (*n*=2) | Semi-structured interview with Experiential Interview Scheme (situation-task-action-result) to initiate discusssion of resilience to adverse experience |
| The personal meanings and experiences of resilience amongst elite badminton athletes in the build up to competition | Adam, R., & Cogan, N. (2019) | Y | Y | Y | Y | Y | 100 | Study delves in-depth with IPA method but did not specify the time-frame of competition build-up. It is also very focused i.e. badminton and retrospective | No guiding theoretical framework | Elite full-time badminton (n=4M, 6F) in National Badminton Center UK | IPA, Semi-structured interview |
| Resilience in Turkish Physically Disabled Athletes: The Role of Sport Participation | Aydogan, D., & Hadi, G. (2020) |  | Y | Y | Y | Y | 100 | Good rigour. However, study conceptualises adversity experiences only as the disability which in too narrow-focused. Resilience is conceptualised as ability to recover after disability and participate in sport which is theoretically unbacked and limited. | Socioecological Framework of Resilience (Ungar, 2008) | 12 (5F 7M) National and International Paralympic Sport Athletes | Resilience Interview Form (RIF) developed based on Ungar (2008) i.e. a interview schedule |
| Overcoming Performance Slumps: Psychological Resilience in Expert Cricket Batsmen | Brown, J., C., Butt, J., Sarkar, M. (2020) |  | Y | Y | Y | Y | 100 | Study provides good practical contributions but does not fully capture the dynamic experience of resilience in play. Potential limitations regarding retrospective bias also noted. | Grounded Theory  of Psychological Resilience  (Fletcher & Sarkar, 2012) | Stage 1 FGD(n=4); Stage 2- Interview (n=10), players who had played a significant amount of professional cricket, County and International | FGD & Semi-structured Interview |
| The personal meanings and expereinces of resilience amongst elite badminton athletes in the build up to competition | Adam, R., & Cogan, N. (2019) |  | Y | Y | Y | Y | 100 | Study delves in-depth with IPA method but did not specify the time-frame of competition build-up. It is also very focused i.e. badminton and retrospective | No guiding theoretical framework | Elite full time badminton (n=4M, 6F) in National Badminton Center UK | IPA, Semi-structured interview |
| Resilience in Turkish Physically Disabled Athletes: The Role of Sport Participation | Aydogan, D., & Hadi, G. (2020) |  | N | Y | Y | Y | 80 | Good rigour. However, study conceptualises adversity experiences only as the disability which in too narrow-focused. Resilience is conceptualised as ability to recover after disability and participate in sport which is theoretically unbacked and limited. | Socioecological Framework of Resilience (Ungar, 2008)Sarkar & Fletcher (2012) and Machida et al (2013) dynamic conceptualisation of resilience with focus on physically disabled individuals | 12 (5F 7M) National and International Paralympic Sport Athletes, 9-23 years of living with disability | Resilience Interview Form (RIF) developed based on Ungar (2008) i.e. a interview schedule |
| Sporting resilience during COVID-19: What is the nature of this adversity and how are competitive elite athletes adapting?. | Gupta & McCarthy (2021) |  | Y | Y | Y | Y | 100 | Two part study which provides thematic representation of COVID advertise and narrative insight into the process of resilience using narrative quest analysis. High resilience individuals screened using BRF (Smith et al., 2008) | Fletcher & Sarkar (2012), Gupta & McCarthy (in press) Model of Sporting Resilience | N= 10 (5F; 5M) competitive elite level athletes from multiple countries including WEIRD countries | Semi-structured, experiential frame interviews |

**Appendix B:** Continued (Mixed Method Studies)

| Study | Authors | Mixed-Method Criteria | | | | | Quality Score | | Notes | Theoretical/Conceptual Framework | Sample | Measures |
| --- | --- | --- | --- | --- | --- | --- | --- | --- | --- | --- | --- | --- |
|  | | C1 | C2 | C3 | C4 | C5 |  |  | |  |  |  |
| The Effects of REBT, and the Use of Credos, on Irrational Beliefs and Resilience Qualities in Athletes | Deen, S., Turner, M. J., & Wong, R. S. (2017) | Y | Y | Y | Y | Y | 100 | Single-case Intervention study, with resilience measures and post intervention social validation | | REBT ABCDE framework (Turner & Barkar, 2014) | Elite national squash athletes (*n*=5) | Connor Davidson Resilience Scale (2003), 10-item; Social Validation using one-on-one semi-structured interviews |
| Resilience, stress and injuries in the context of the Brazilian elite rhythmic gymnastics | Codonhato, R., Rubio, V., Oliveira, P. M. P., Resende, C. F., Rosa, B. A. M., Pujals, C., & Fiorese, L. (2018). | Y | N | Y | Y | Y | 80 | Sequential Explanatory Design, Longitudinal study. However, due to high specificity and limts of sample, generalisation is limited | | Grounded Theory of Psychological Resilience (Fletcher & Sarkar, 2012) | Female Brazilian Olympic Rhythmic gymnasts during prep period of Rio Olympics (*n*=8) | Quantitative- Connor Davidson Resilience Scale (2003), 10-item, documental analysis of physical therapy records. Qualitative- structured questionnaire with open ended questions based on Fletcher & Sarkar (2012) |
| A Mixed Methods Evaluation of a Pressure Training Intervention to Develop Resilience in Female Basketball Players | Kegelaers, J., Wylleman, P., Bunigh, A., & Oudejans, R. R. (2019) | Y | Y | Y | Y | Y | 100 | Mixed methods evaluation of intervention efficacy. No time series data and limited to females. | | Mental Fortitude Training Theory (Fletcher & Sarkar, 2016a) | Elite female basketball players (*n*=19) | Quantitative- Connor Davidson Resilience Scale (2003), 10-item and Team Resilience Scale (Decroos et al.,2017); Qualitative-three part semi-structured interviews |
| The Efficacy of a Resilience Intervention Among Diverse, At-Risk, College Athletes: A Mixed-Methods Study | Chandler, G., Kalmakis, A., K., Chiodo, L., & Helling, J. (2020) | Y | N | Y | Y | Y | 80 | Mixed methods evaluation of a ABCS Intervention among ACE and at-risk student athlete population. Control group very small, no random assignment and no Post Intervention data | | SDT & CMCL ABCS Resilience Framework | Division 1 Football (n=47) & Women's basketball (n=15) | Quantitative- CDRISC-25, Qualitative- In class reflections notes and presentations |

**Appendix B:** Continued (Review Studies)

| Study | Authors | Review Study Criteria | | | | | | | | | | Quality Score | Notes |
| --- | --- | --- | --- | --- | --- | --- | --- | --- | --- | --- | --- | --- | --- |
|  | | C1 | C2 | C3 | C4 | C5 | C6 | C7 | C8 | C9 | C10 |  |  |
| How Should we Measure Psychological Resilience in Sport Performers? | Sarkar, M. & David Fletcher, D. (2013) | Y | Y | Y | Y | Y | Y | Y | Y | Y | Y | 100 | Provides important insight into psychometric and other measurement issues of resilience in sport |
| Psychological resilience in sport performers: a review of stressors and protective factors | Sarkar, M., & Fletcher, D. (2014) | Y | Y | N | Y | Y | Y | Y | Y | Y | Y | 90 | Highlights limitations and successes of extant literature while providing a comprehensive review at the time, listing out future directions for research |
| Psychological resilience in sport: A review of the literature and implications for research and practice | Galli N., & Gonzalez S. (2015) | Y | Y | N | Y | Y | Y | Y | Y | Y | Y | 90 | Highlights limitations and successes of extant literature while providing a comprehensive review at the time, listing out future directions for research |
| Resilience In Sports A critical review of psychological processes, sociocultural influences, and organisational dynamics | Wagstaff, C. R., Sarkar, M., Davidson, C. L., & Fletcher, D. (2016) | Y | Y | Y | Y | Y | Y | Y | N | Y | Y | 90 | Highlights the organisational component of resilience noting the socioecological components at play for resilience in sports contexts which make it unique. |
| Stressing the relevance of resilience: a systematic review of resilience across the domains of sport and work | Bryan C., O'Shea D., & MacIntyre T. (2019) | Y | Y | N | Y | Y | Y | Y | Y | Y | Y | 90 | Systematic review focused on resilience in the domains of both work and sport and as such does not include all available literature of resilience in sport. A notable limitation is the lack of inclusion of studies from non-western contexts and of the topical area of team resilience nor are conceptual contributions such as book chapters/theoretical articles included. The synthesised definition provided is not sport-specific and does not capture the unique complexities of a sporting context. |
| Resilience of athletes: A systematic review based on a citation network analysis | Bicalho, C.C.F., Melo, G.F., Noce, F.(20200 | Y | Y | Y | Y | Y | Y | Y | N | Y | N | 80 | Systematic review using citation network analysis gives an insight into the trends of research and citation map. The majority of the research conducted is guided by Fletcher & Sarkar (2012). This provides an important cause for reflection since findings may be overtly confirmatory due to the base of one major theoretical perspective. |

**Appendix B:** Continued (Conceptual/Theoretical Studies)

| Study | Authors | Conceptual/Theoretical  Criteria | | | | | Quality Score | Notes |
| --- | --- | --- | --- | --- | --- | --- | --- | --- |
|  | | C1 | C2 | C3 | C4 | C5 |  |  |
| A Protocol for Teaching Resilience to High Performance Athletes | Schinke, R.J., Peterson, C., & Couture, R. (2004) | Y | Y | Y | Y | Y | 100 | Resilience development via improved cognitive and emotional control |
| Fostering Self-Determination and Resilience Through Sports Counselling: Psychosocial Development of Youth in Botswana | Tinsley, T.M., & Levers, L.L (2007) | Y | Y | Y | Y | Y | 100 | Culturally sensitive athletic programs and sports counselling based on using SDT principles to foster resilience in youth |
| Case studies of developing resilience in elite sport: Applying theory to guide interventions | Gonzalez, S. P., Detling, N., & Galli, N. A. (2016). | Y | Y | Y | Y | Y | 100 | Provides a framework with components characterising development and demonstration of resilience |
| Proposing a rational resilience credo for use with athletes | Turner, M., J. (2016) | Y | Y | Y | Y | Y | 100 | REBT based resilience enhancement intervention |
| Developing Resilience Through Coaching | Sarkar, M., & Fletcher, D. (2016b) | Y | Y | Y | Y | Y | 100 | Resilience training via coaching and coach-athlete relationship via psychosocial training and developmental experiences. |
| Mental fortitude training: An evidence-based approach to Developing psychological resilience for sustained success | Fletcher, D., & Sarkar, M. (2016a) | Y | Y | Y | Y | Y | 100 | Focus on personal qualities, facilitative environment and challenge mindset as key to resilience. |
| Team Resiliency in Sport research to practice | Yukelson, D., & Weinberg, R. (2016) | Y | Y | Y | Y | Y | 100 |  |
| PasSport4life: A trainee sport psychologist's perspective on developing a resilience-based life skills program | Cox, H., Neil, R., Oliver, J., & Hanton, S. (2016). | Y | Y | Y | Y | Y | 100 | Novel intervention employing life skills-A pilot programme. |
| Team Resilience | Galli, N. (2016) | Y | Y | Y | Y | Y | 100 | Provides a socioecological perspective on team resilience |
| Resilience in Sports from a Dynamical Perspective | Hill, Y., den Hartigh, R.J., Peter de Jonge, R.R.M., & Van Yperen, N.W. (2018a) | Y | Y | Y | Y | Y | 100 | Proposes a novel theoretical model highlighting resilience process |
| The What, How, Where and When of Resilience as a Dynamic, Episodic, Self-Regulating System: A Response to Hill et al. (2018) | Bryan, C., O'Shea, D., & MacIntyre, T. E. (2018). | Y | Y | Y | Y | Y | 100 | Response to Hill et al(2018a) |
| Furthering the Discussion on the Use of Dynamical Systems Theory for Investigating Resilience in Sport | Galli, N., & Pagano, K. (2018) | Y | Y | Y | Y | Y | 100 | Response to Hill et al(2018a) |
| The Temporal Process of Resilience | Hill, Y., den Hartigh, R.J., Peter de Jonge, R.R.M., & Van Yperen, N.W. (2018b) | Y | Y | Y | Y | Y | 100 | Response to Hill et al(2018a) |
| Psychological resilience and adversarial growth in sport and performance. | Fletcher, D. (2019) | Y | Y | Y | Y | Y | 100 | Conceptual advancements in relationship of resilience and adversity and process of resilience |

**Appendix C**

Definitions of Resilience in included studies

| Articles reporting their own definition of resilience | |
| --- | --- |
|  |  |
| Holt and Dunn (2004) | ‘The term resilience is used here to reflect the ability to bounce back after adversity’ |
| Nezhad and Besharat (2010) | ‘Resilience generally refers to an individual capacity in the face of stressful events and a pattern of functioning indicative of positive adaptation in context of risk or adversity, underlying two conditions: (a) exposure to risk and (b) positive adaptation.’ |
| Fletcher and Sarkar (2012) | “the role of mental processes and behaviour in promoting personal assets and protecting an individual from the potential negative effect of stressors” |
| Belem et al. (2014) | ‘A psychological characteristic that explains the favourable responses of athletes during competitions even after they had experienced adverse circumstances’ |
| Vitali et al. (2015) | ‘Resilience is thought as a personal trait that enables an individual to thrive in the face of adversity.’ |
| Secades et al., (2016) | ‘Resilience can be defined as the individual's skill of keeping performance levels relatively stable and it can cause positive adaptation in response to significant adversities experiences by a given person' |
| Bryan et al., (2017) | ‘A dynamic process encompassing the capacity to maintain regular functioning through diverse challenges or to rebound through the use of facilitative resources’ |
| Hill et al., (2018b) | Resilience to be ‘the dynamic process by which a biopsychosocial system returns to the previous level of functioning following a perturbation caused by a stressor’ |
| Kegelaers et al., (2019) | Resilience is the dynamic process of mobilizing protective factors in order to reach or maintain a positive outcome despite exposure to stress or adversity' |

**Appendix C:** Definitions of Resilience in included studies

| Articles citing guiding definitions of resilience | | |
| --- | --- | --- |
| **Definition Source Article** | **Definition of resilience** | **Cited by (author/year)** |
| Carver and Schieier (2002) | ‘The ability to “bounce back” from stressful experiences quickly and efficiently’ | Subhan and Ijaz (2012) |
| Fletcher and Sarkar (2012) | ‘The role of mental processes and behaviour in promoting personal assets and protecting an individual from the potential negative effect of stressors’ | Sarkar and Fletcher (2014); Galli and Gonzalez (2015); Gonzalez et al., (2016); Kegelaers et al., (2019); Lu et al. (2016); Meggs et al. (2016); Turner (2016); Sarkar and Fletcher (2016); Secades et al. (2016); Wagstaff et al., (2016); Belem et al., (2017); Timm et al., (2017); Sarkar (2017); Yamada et al., (2017); Kegelaers and Wylleman (2018); Wagstaff et al., (2018); Trigueros et al., (2019); Brown et al., (2020); Pedro (2016); Drew & Matthews (2018); Sullivan et al., (2021) |
| Galli and Vealey (2008) | ‘An athlete’s ability to positively adapt and develop resilient qualities in the presence of adversity' | Brown et al. (2015); Belem et al., (2017) |
| Luthar et al. (2000) | ‘Dynamic process encompassing positive adaptation within the context of significant adversity' | Galli and Vealey (2008); Hosseini and Besharat (2010); Gucciardi et al. (2011); Fletcher and Sarkar (2012);; Machida et al. (2013); Sarkar and Fletcher (2013); Gonzalez et al. (2016); Sarkar and Fletcher (2016); Neves et al., (2017); Codonhato et al., (2018); |
| Masten (1994) | ‘The process of, capacity for, or outcome of successful adaptation despite challenging circumstances' | Galli and Vealey (2008) |
| Masten (2001) | ‘An individual’s capacity to maintain psychological and physical well-being in the face of adversity' | Yi et al. (2005); Hosseini and Besharat (2010) |
| Rutter (1987) | ‘The ability to bounce back or cope successfully despite substantial adversity' | Mummery et al., (2004); Adam & Cogan (2019) |
| Rutter (1993) | ‘individual’s ability to face, overcome and grow from life’s adversities’ | Gonzalez et al., (2020) |
| Wagnild and Young (1993) | ‘resilience is defined as a trait that decreases the negative effects of stressful situation enables a successful accommodation and eluding negative consequences’ | Bingol & Bayansalduz (2016) |
| Windle et al. (2011) | ‘An outcome or demonstration of positive adaptation following exposure to adversity or risk, as a result of the availability and use of internal and external resources’ | Cardoso and Sacomori (2014); Cowden and Meyer-Weitz (2016) |
| Wald et al., (2006) | Resilience is the capacity of the individual to bounce-back after experiencing stress | Gupta and Sudhesh (2019) |
| Connor and Davidson (2003) | Resilience embodies the personal qualities that enable one to thrive in the face of adversity' | Neves et al., (2017); Chacon-Cuberos et al., (2019) |
| Tugade and Fredrickson (2004) | Resilience is an ability to withstand and rebound from disruptive life challenges' | Young (2014) |
| Fletcher and Sarkar (2016) | Resilience refers to the ability to use personal qualities to withstand pressure' | Sarkar (2018) |
| Morgan et al., (2013) | Team Resilience is defined as a dynamic, psychosocial process which protects a group of individuals from the potential negative effect of the stressors they collectively encounter' | Morgan et al., (2015; 2019); Galli (2016); Yukelson and Weinberg (2016); Decroos et al., (2017); |
| Secades et al., (2016) | Resilience can be defined as the individual's skill of keeping performance levels relatively stable and it can cause positive adaptation in response to significant adversities experiences by a given person' | Secades et al., (2017) |
| Dryden (2011) | ‘Resilience comprises a set of flexible cognitive, behavioural and emotional responses to acute or chronic adversities that can be unusual or commonplace.' | Deen et al., (2017) |
| Masten, Best and Garmezy (1990) | the process of, capacity for, or outcome of successful adaptation despite challenging or threatening circumstances' | Galli and Gonzalez (2015); Ueno and Suzuki (2016); Gupta and Sudhesh (2019) |
| Bryan et al., (2017) | ‘A dynamic process encompassing the capacity to maintain regular functioning through diverse challenges or to rebound through the use of facilitative resources’ | Bryan et al., (2018) |
| Lynch and Levers (2007) | Broadly speaking, resilience refers to positive or adaptive developmental outcomes despite the presence of risk factors' | Tinsley and Levers (2007) |
| Castro et al., (2016) | ‘The ability to overcome adverse situations' | Zurita-Ortega et al., (2016) |
| Ishige and Muto (2005) | ‘Resilience is the mental ability to recover from negative psychological states caused by stressful incidents' | Ueno and Oshio (2017) |
| Forés and Grané (2008) | ‘resilience as a set of personal qualities that constitute human ability to overcome adverse and stress situations. At the same time, it leads to positive growth of the individual as a result of facing their own personal and sporting challenges’ | Trigueros et al., (2020a) |
| Gupta and McCarthy (2021) | The environmentally adaptable, interaction dominant, dynamic-process trajectory that encompasses a sporting individual’s metacognitive–emotional–behavioral capacities to maintain a positive equilibrium and successfully adapt to a diverse range of sport-related adversities | Madsen et al., (2021); Martinez-Gonzalez et al., (2021) |
| Luthans (2002) | Resilience is the rapid recovery of the individual after a negative event | Kilic (2021) |

Note: No guiding definitions were cited by Smith et al., (1990); Seligman et al., (1990); Martin-Krumm et al., (2003); Schinke et al., (2004); Hall (2011); Bejan and Tonita (2014); White and Bennie (2015); Cox et al., (2016); Ruiz-Barquin et al., (2017); Metin et al., (2017); Ortega et al., (2018); Codonhato et al., (2018); Sorkkila et al., (2019); Fletcher (2019); Ozdemir (2019); Karademir & Gencay (2020); Aydogan & Hadi (2020); Trigueros et al., (2020b); Martin et al., (2015); Aydogan & Hadi (2020); McGivern et al., (2021)
